# Male grasshoppers (*Glyptobothrus maritimus*) in roadside habitats have increased stridulatory sound-producing organs

**DOI:** 10.1038/s42003-026-10181-4

**Published:** 2026-05-05

**Authors:** Jou Yoshigai, Keisuke Nakaoka, Tomoki Hirose, Ryugo Ishida, Daiki Waga, Takehiko Shimizu, Masayuki Senzaki

**Affiliations:** 1 Graduate School of Environmental Science, Hokkaido University, Sapporo, Japan; 2 Graduate School of Agriculture, Hokkaido University, Sapporo, Japan; 3 Faculty of Environmental Earth Science, Hokkaido University, Sapporo, Japan

**Keywords:** Conservation biology, Behavioural ecology, Entomology

## Abstract

Anthropogenic noise disrupts animal communication, often prompting shifts toward higher-frequency signals to avoid masking by low-frequency noise. While such changes are typically attributed to behavioral plasticity in vocal species, this may not apply to non-vocal sound producers. We tested whether male grasshoppers (*Glyptobothrus maritimus*) exhibit morphological changes in response to noise by examining stridulatory organ density and courtship signal frequencies across habitats with varying traffic noise. In the first survey year, grasshoppers from the noisiest habitats had 13.4% higher organ density—a trait that may favor higher-frequency signals—than those from the quietest habitats. However, this difference became unclear in the following year. Additionally, courtship signal frequencies showed no positive relationship with organ density or noise. These findings suggest that elevated sound levels can induce short-term developmental processes that generate variation in sound-producing organs in non-vocal animals, underscoring how neglecting morphological plasticity, independent of signal modification, constrains understanding of ecological noise impacts.

## Abstract (web summary)

Traffic noise increases the density of stridulatory organs in male grasshoppers, likely through short-term developmental processes, but does not elevate courtship signal frequency.

## Introduction

The emission of anthropogenic noise has been rapidly increasing worldwide over the past few decades^1,2^. Previous research has demonstrated that noise impairs animal behavior^3^, physiology^4^, and reproduction^5^ as well as population dynamics^6^, community structures^7^, and ecosystem functioning^8^. Among these, the most well-known ecological effects of noise pollution are the reduced efficacy of transmission and reception of biologically relevant sounds in noise^9^, known as acoustic masking. This adverse effect of noise has been documented in various terrestrial animals, including birds^10^, bats^11^, amphibians^12^, and insects^13^. Moreover, these animals have been demonstrated to elevate their signal frequencies to reduce the masking effects from typical low-frequency noise^9,12–14^.

Previous research has assumed that behavioral plasticity would underlie such frequency shifts in various animals, particularly those capable of vocal sound production^15,16^. However, this mechanism alone may not account for all the documented frequency changes, especially in species producing non-vocal sounds^13^. For instance, grasshoppers produce courtship signals by stridulating their sound-producing organs, and this necessary behavior may constrain their ability to flexibly modulate signal frequencies in noisy environments^17–19^. While these species may retain some behavioral plasticity, this potentially limited ability points to a largely overlooked hypothesis that species relying on non-vocal sound production may respond to louder acoustic environments through morphological changes in their sound-producing organs. Addressing this underappreciated hypothesis is critical for revealing the full extent of the ecological costs of noise pollution and understanding how animals can/cannot cope with this widespread environmental stressor.

Here, we aim to test the hypothesis that species relying on non-vocal sound production exhibit morphological differences in their sound-producing organs in response to noise exposure. To this end, we compared morphology of the sound-producing organs of a grasshopper species along a gradient of traffic noise exposure for the following reasons: (1) grasshoppers are commonly found in roadside grasslands and are frequently exposed to traffic noise, a major source of terrestrial anthropogenic noise^20,21^; (2) many grasshopper species rely on acoustic communication during reproduction, and their courtship signal frequencies often overlap with traffic noise^13^; (3) previous studies have reported that grasshoppers can increase the frequency of their courtship signals in response to noise^13,22^; and (4) artificially reared *Chorthippus biguttulus* from noisy roadside habitats produce higher-frequency courtship signals than those from quiet areas, even when raised under quiet conditions^23^, suggesting that morphological differences may underlie this frequency shift.

We conducted field sampling in grasslands that were either quiet or exposed to varying levels of traffic noise in five major regions in Hokkaido, northern Japan. At each sampling site, we collected 7–22 males of *Glyptobothrus maritimus*, the most common grasshopper species in the region, and belonging to the same subfamily (Gomphocerinae) as the well-studied *C. biguttulus*^13,23^. We examined how the density of stridulatory file teeth—the primary sound-producing structures—was associated with traffic noise exposure. We also examined whether these morphological traits and levels of noise exposure were associated with courtship signal frequency. We predicted that males from noisier habitats would possess a higher density of stridulatory teeth, a trait likely linked to increased signal frequency, and hence produce courtship signals at higher frequencies compared to those from quieter habitats.

## Results

### Acoustic conditions

**Fig. 1:**
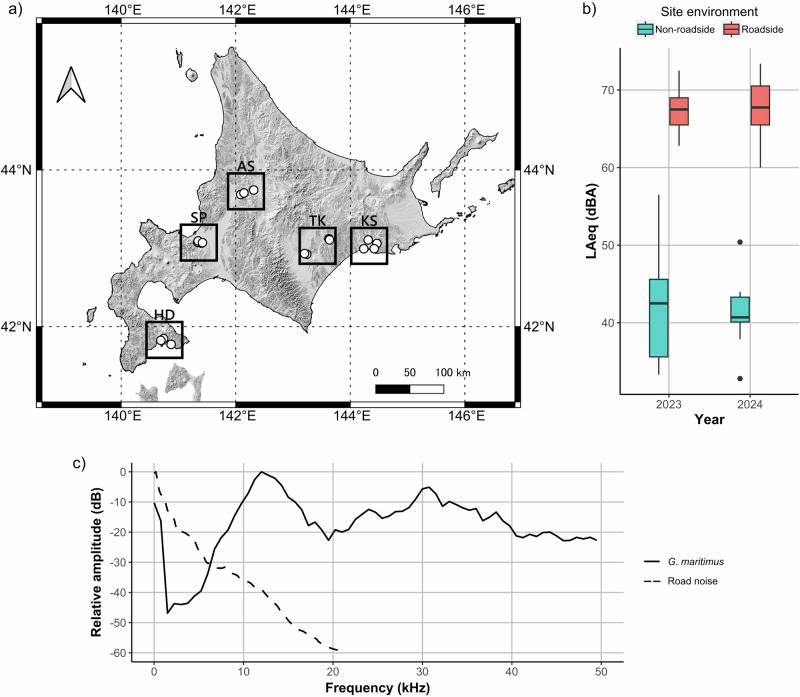
Study area and acoustic measurement results. **a** Map of Hokkaido Island with the study regions (black rectangles) and the study sites (white circles). Abbreviations above the rectangles indicate the names of the regions. The base map was obtained from the Geospatial Information Authority of Japan website (https://maps.gsi.go.jp/development/ichiran.html). **b** Noise level comparison between the acoustic environments of the study sites across the years. The box plots show distributions of LAeq (dBA) measured in each site and year. **c** Power spectra of a male *G. maritimus* courtship signal and traffic noise. The courtship signal was recorded on a male collected at the site HU during the summer of 2025. The recording was conducted in a soundproof chamber at Hokkaido University. The traffic noise was recorded at the site NT in 2023. The peak frequency of this species typically appears between 10 and 15 kHz, which is more likely masked by traffic noise than the higher frequency range.

Noise measurements showed that 5-min equivalent continuous A-weighted sound pressure levels (LAeq) varied among sampling sites, and roadside habitats were exposed to a relatively wide range of traffic noise (60.0–73.4 dBA, Supplementary Table S1, Fig. 1). More specifically, the mean LAeq [5 min] (±SD) values were 41.5 ± 5.59 in non-roadside habitats and 67.4 ± 3.11 dBA in roadside habitats (Fig. 1).

### Morphological traits in response to noise

**Fig. 2:**
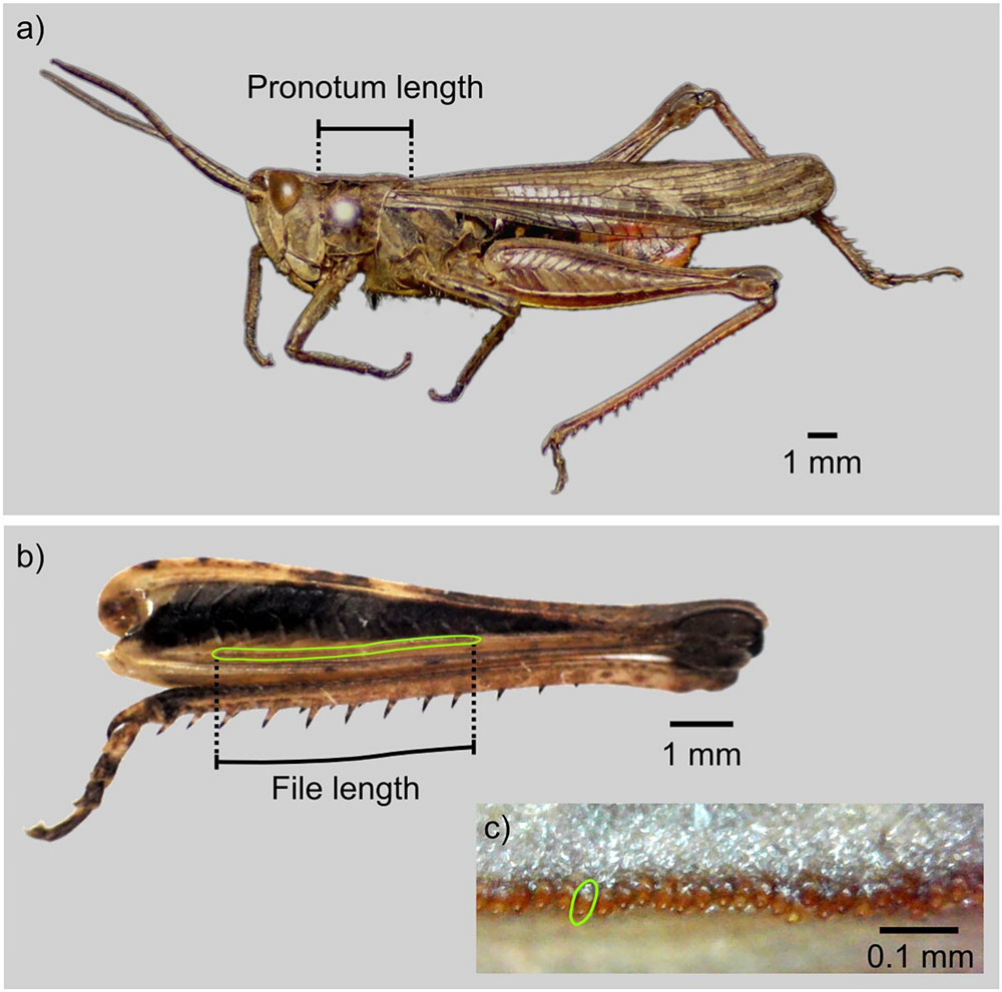
Morphology of a male *G. maritimus.* **a** Lateral view. The black solid bar indicates the measurement range for pronotum length. **b** Inner side of a right hind leg. The region outlined in green indicates the position of a stridulatory file, and the black solid bar represents file length. **c** Close-up image of file teeth. The green-lined enclosures show a single tooth.

We collected a total of 299 intact male *G. maritimus* individuals (Fig. 2). The mean pronotum length, file tooth density, file length, and number of file teeth (±SD) were 3.29 ± 0.02 mm, 31.50 ± 3.44 teeth/mm, 8.07 ± 0.04 mm, and 253.73 ± 28.59 teeth, respectively.

**Fig. 3:**
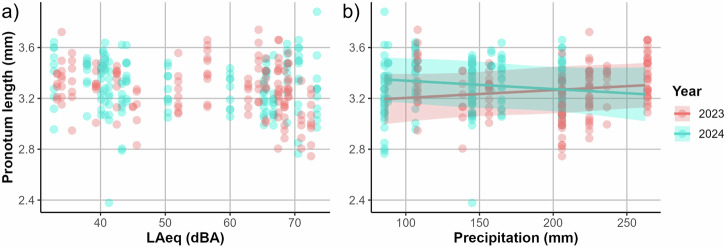
Results of the generalized linear mixed model (GLMM) for pronotum length. Relationships between pronotum length and **a** LAeq and **b** Precipitation. See also Table 1 for specific estimates in the GLMM. Pronotum length was not associated with LAeq values. Lines in (**b**) show the predicted curves fitted by the GLMM, and color bands exhibit these 95% CIs. Points show the individual-level raw data. Different colors represent samples in different years (Red: 2023, Blue: 2024).　Precipitation is represented as the sum of average daily precipitation between May 1 and July 15.

**Fig. 4:**
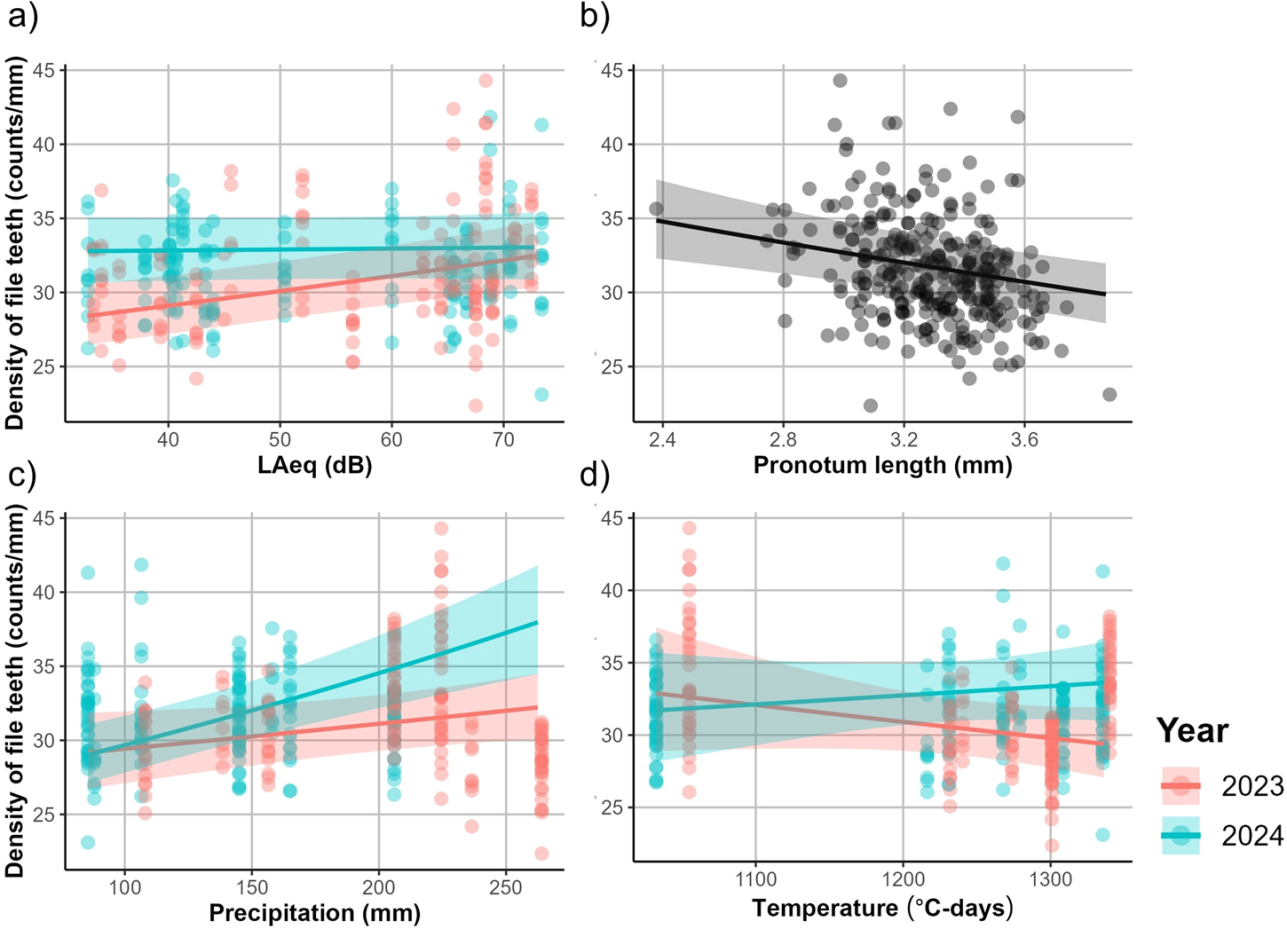
Results of the GLMM for file tooth density. Relationships between file tooth density and physiological factors (**a**–**d**). Significant results in the GLMM were shown. See also Table 1 for specific estimates in the GLMM. The effects of **a** LAeq, **c** precipitation, and **d** temperature were different between years, whereas the effect of **b** pronotum length was consistent between years. Lines show the predicted curves fitted by the GLMM, and color bands exhibit these 95% CIs. Points show the individual-level raw data. Different colors for (**a**, **c**, **d**) represent samples in different years (Red: 2023, Blue: 2024). Precipitation or temperature is represented as the sum of average daily precipitation or temperature between May 1 and July 15.

Pronotum length was not associated with any of the environmental variables tested, except for the interaction between year and precipitation (Table 1, Fig. 3), indicating similar variation in this trait across habitats with varied acoustic conditions. In contrast, file tooth density increased with increasing LAeq values; it was 13.4% higher in the noisiest habitats than in the quietest habitats, supporting our prediction (Table 1, Fig. 4). However, the interaction between survey year and LAeq indicated that this noise-related increase in file tooth density was evident in 2023 but not in 2024 (Table 1, Fig. 4). File tooth density increased with pronotum length and precipitation, with the effect of precipitation being stronger in 2024, and was higher in 2024 than in 2023 (Table 1, Fig. 4). Temperature showed no overall effect on file tooth density, but was positively associated with density in 2024 (Fig. 4, Table 1).

**Table 1:** Results of GLMMs testing the effects of acoustic environments on morphological and acoustic traits in *G. maritimus*

Response variable	Explanatory variable (Random effect)	Estimate	SE	95% CI	*P*-value	*R*²	KS	Dispersion	Outlier
Pronotum length	**(Intercept)**	**3.25**	**0.06**	**[3.13, 3.36]**	**0.00**	0.39 (0.04)	0.61	0.6	1
	LAeq	−0.02	0.02	[−0.07, 0.02]	0.32				
	Temperature	−0.03	0.06	[−0.14, 0.09]	0.67				
	Precipitation	0.04	0.03	[−0.03, 0.10]	0.27				
	Year	0.05	0.04	[−0.03, 0.13]	0.23				
	Temp:Year	0.00	0.02	[−0.04, 0.05]	0.87				
	**Precip:Year**	**−0.07**	**0.03**	**[−0.13, 0.01]**	**0.02**				
	LAeq:Year	0.03	0.02	[−0.02, 0.07]	0.27				
	(Site ID)	0.08							
	(Region ID)	0.11							
File teeth density	**(Intercept)**	**3.42**	**0.03**	**[3.36, 3.48]**	**0.00**	0.57 (0.28)	0.56	0.59	0.98
	**LAeq**	**0.05**	**0.01**	**[0.03, 0.07]**	**0.00**				
	**Pronotum length**	**−0.02**	**0.01**	**[−0.03, −0.01]**	**0.00**				
	Temperature	−0.04	0.03	[−0.10, 0.02]	0.18				
	**Precipitation**	**0.03**	**0.02**	**[0.00, 0.06]**	**0.04**				
	**Year**	**0.07**	**0.02**	**[0.03, 0.11]**	**0.00**				
	**Temp:Year**	**0.06**	**0.01**	**[0.04, 0.09]**	**0.00**				
	**Precip:Year**	**0.05**	**0.01**	**[0.03, 0.08]**	**0.00**				
	**LAeq:Year**	**−0.04**	**0.01**	**[−0.06, −0.02]**	**0.00**				
	(Site ID)	0.02							
	(Region ID)	0.07							
File length	**(Intercept)**	**7.82**	**0.13**	**[7.56, 8.08]**	**0.00**	0.56 (0.33)	0.46	0.49	1
	LAeq	−0.12	0.07	**[−0.25, 0.01]**	0.07				
	**Pronotum length**	**0.16**	**0.03**	**[0.10, 0.22]**	**0.00**				
	Temperature	0.04	0.13	**[−0.21, 0.31]**	0.74				
	Precipitation	−0.11	0.08	**[−0.27, 0.04]**	0.15				
	**Year**	**0.48**	**0.10**	**[0.26, 0.68]**	**0.00**				
	**Temp:Year**	**−0.13**	**0.06**	**[−0.25, 0.02]**	**0.03**				
	Precip:Year	0.00	0.08	**[−0.15, 0.16]**	0.99				
	**LAeq:Year**	**0.12**	**0.06**	**[0.01, 0.24]**	**0.04**				
	(Site ID)	0.22							
	(Region ID)	0.24							
Number of file teeth	**(Intercept)**	**5.50**	**0.01**	**[5.47, 5.52]**	**0.00**	0.30 (0.23)	0.68	0.85	0.96
	**LAeq**	**0.03**	**0.01**	**[0.01, 0.05]**	**0.01**				
	Pronotum length	0.00	0.01	[−0.02, 0.01]	0.52				
	**Temperature**	**−0.05**	**0.01**	**[−0.07, −0.02]**	**0.00**				
	Precipitation	−0.01	0.01	[−0.04, 0.01]	0.31				
	**Year**	**0.09**	**0.02**	**[0.06, 0.13]**	**0.00**				
	**Temp:Year**	**0.05**	**0.01**	**[0.02, 0.07]**	**0.00**				
	**Precip:Year**	**0.04**	**0.02**	**[0.01, 0.07]**	**0.01**				
	**LAeq:Year**	**−0.03**	**0.01**	**[−0.05, −0.00]**	**0.03**				
	(Site ID)	0.03							
	(Region ID)	0.00							
Peak frequency	**(Intercept)**	**9242.75**	**191.78**	**[8866.10, 9619.40]**	**0.00**	0.37 (0.07)	0.06	0.73	0.49
	LAeq	82.23	145.08	[−202.72, 367.18]	0.57				
	**Year**	**898.99**	**157.16**	**[590.34, 1207.64]**	**0.00**				
	**Pronotum length**	**−160.13**	**76.74**	**[−310.85, −9.41]**	**0.04**				
	Tooth density	34.81	72.28	[−107.15, 176.77]	0.63				
	LAeq:Year	16.36	156.50	[−291.00, 323.72]	0.92				
	(Individual ID)	724.18							
	(Site ID)	275.82							
	(Region ID)	275.84							

We analyzed the effect of noise on morphological traits from 299 intact males. While we obtained acoustic trait data from 276 males, we excluded outliers from 7 individuals and analyzed the effect of noise on acoustic traits from 269 males. Bold values indicate 95% confidence intervals that do not include zero, suggesting statistical significance. In the “Explanatory variable” column, “Temp:Year”, “Precip:Year”, and “LAeq:Year” indicate the interaction terms between year and temperature, precipitation, and LAeq, respectively. Estimates of random effects are shown as standard deviations. In the “*R*^2^” column, values outside parentheses indicate conditional *R*^2^, whereas those in parentheses indicate marginal *R*^2^. “KS”, “Dispersion”, and “Outlier” refer to the results of model diagnostics for the Kolmogorov-Smirnov test, overdispersion test, and outlier test, respectively. Values in these diagnostic columns represent *P*-values.

Because file tooth density reflects the combined effects of file length and the number of file teeth, we examined how these two components varied across noise gradients. In 2023, the number of file teeth increased, whereas file length decreased in noisier habitats in 2023 (Table 1), suggesting that concurrent changes in both traits could contribute to the higher file tooth density associated with noise in that year.

### Acoustic traits in response to noise

Among 299 individuals collected, we obtained high-quality recordings of the peak frequency of courtship signals from 276 individuals, and the mean peak frequency (±SD) was 10023.37 ± 1595.56 Hz.

**Fig. 5:**
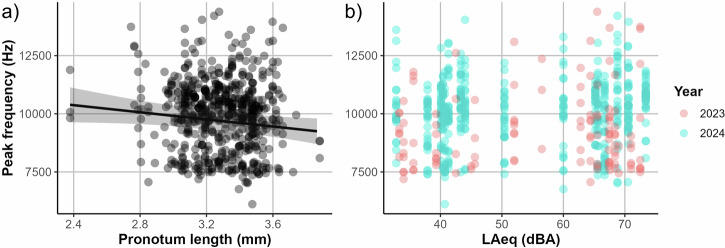
Results of the GLMM for peak frequency. Relationships between peak frequency and **a** pronotum length and **b** LAeq. Peak frequency was not associated with LAeq values. See also Table 1 for specific estimates in the GLMM. The line and band in (**a**) show the predicted curve and 95% CI fitted by the GLMM. Points show the individual-level raw data. Different colors in (**b**) represent samples in different years (Red: 2023, Blue: 2024).

In contrast to our prediction of the relationship between file tooth density and acoustic signaling, peak frequency was not associated with the density of file teeth, LAeq, and their interaction (Table 1, Fig. 5), suggesting no clear elevation of courtship signals along a gradient of noise exposure in our model species. This acoustic trait was positively and negatively associated with year and pronotum length, respectively (Table 1, Fig. 5). That is, individuals in 2023 produced higher frequency signals than those in 2024, and larger individuals produced lower frequency signals than smaller individuals.

## Discussion

As expected, male grasshoppers inhabiting noisier environments possessed higher file tooth density than those from quieter habitats. However, this relationship was evident in only one of the two survey years. Moreover, contrary to our expectation and findings from previous studies^13,22,23^, we found no consistent difference in courtship signal peak frequency across noise gradients, nor was variation in signal frequency explained by file tooth morphology among habitats.

The higher file tooth density in noisy habitats in the first year was consistent with our hypothesis. However, the temporal inconsistency between the two survey years strongly suggests that the observed differences in sound-producing organs along a noise exposure gradient would not be explained by stable genetic differentiation among populations. These patterns are instead consistent with developmental plasticity induced by short-term environmental variation during development, such as temperature or nutrition^24^. Developmental plasticity can rapidly alter morphology, physiology, and behavior in response to environmental conditions^25,26^. Although roadside noise is generally chronic, our results suggest that interannual variation in developmental conditions may alter the sensitivity of sound-producing organs during critical periods of development, potentially modulating plastic responses to chronic noise exposure.

Importantly, plastic developmental phenotypes are not always adaptive, especially in stressful or suboptimal environments^27^. Under this scenario, the observed increase in file tooth density may be attributed to a maladaptive byproduct of developmental stress rather than a trait shaped by direct selection on acoustic signaling. This interpretation would be supported by the absence of any corresponding elevation in courtship signal frequency in roadside grasshoppers, despite previous studies showing that stridulatory morphology can be a key factor in determining acoustic traits, including signal frequency^28,29^. This expectation arises because an upward frequency shift in acoustic signaling in response to increased file tooth density would be expected if this morphological change was functionally optimized to improve signal transmission in noisy environments^19,28,29^. Instead, the observed mismatch between morphological and acoustic traits suggests that increased investment in sound-producing organs may impose energetic or developmental costs without providing clear reproductive benefits.

Nevertheless, other possibilities demonstrated in other insects may explain the observed increases in file tooth density of roadside grasshoppers. In general, acoustic traits and their associated morphology are often governed by complex genetic architectures and multiple developmental pathways^30,31^. This suggests that similar phenotypes could arise through different genetic or developmental routes, potentially explaining the observed increases in file tooth density. Moreover, variation in traits, such as file tooth density, may affect acoustic properties in ways not directly reflected in peak frequency, including temporal structure, amplitude, and mechanical efficiency of stridulation^32,33^. Beyond these acoustic effects, increased file tooth density may also serve as a non-acoustic function^33,34^. Population genetic drift in isolated roadside populations, indirect selection on correlated traits, developmental constraints on acoustic organ evolution, and environmental effects on resource allocation during development are all plausible factors that deserve future research attention^34,35^.

Alternatively, differences in signal frequency along a noise gradient may not have been expressed or detected because our acoustic recordings were conducted under ambient conditions. In this context, the observed morphological difference may represent an adaptive response to noise exposure that facilitates context-dependent plasticity in acoustic signaling, whereby individuals adjust signal frequency according to noise levels. For example, flexible stridulatory movement speeds could modify signal frequency. Although this interpretation challenges the prevailing assumption that grasshoppers have limited behavioral flexibility in modulating acoustic signals^17–19^, it is consistent with well-documented shifts toward higher signal frequencies across diverse taxa as a strategy to reduce acoustic masking and enhance reproductive success in noisy environments^13,36,37^. Empirical testing of this hypothesis will require acoustic data collected under controlled noise-exposure conditions.

In contrast to our finding of no elevated courtship signal frequency in noisy habitats, previous studies have reported increases in signal frequency in several grasshopper species exposed to anthropogenic noise, including *Chorthippus biguttulus*, a close relative of our study species^13,23^. Differences in phylogenetic, evolutionary, ecological, and morphological factors between the species, as well as in statistical approaches, may contribute to the discrepancy. In addition, species-specific differences in body size (e.g., pronotum length) and signal frequency characteristics may account for this discrepancy. Specifically, our species exhibits a shorter pronotum length and inherently higher peak frequencies in its courtship signals compared to *C. biguttulus*^13,23^. These morphological and acoustic traits may render the signals of our species more resistant to the masking effects of low-frequency-dominated traffic noise, potentially explaining the lack of detectable frequency shifts in populations exposed to traffic noise. Moreover, the observed negative association between peak frequency and pronotum length in our species suggests that lower-frequency signals may serve as honest indicators of male quality, consistent with previous studies showing females may prefer male signals containing both high and low frequency components^38^. If this is the case, a potential trade-off between noise-induced increases in signal frequency and female preference could constrain upward frequency modulation under noisy conditions. Empirical testing of this hypothesis would require experimental assessment of female preferences for courtship signals across a range of frequencies under noise exposure.

This study has four potentially important limitations that merit future research attention. First, prior research suggests that only a subset of stridulatory file teeth may contribute to the peak frequency of courtship signals^19^, suggesting that our focus on the file tooth density may underestimate noise-induced morphological effects. Second, acoustic properties, such as the peak frequency of courtship signals, may be influenced not only by the total number and density of stridulatory file teeth, but also by other factors, including the speed of stridulatory motion, the height of individual teeth, the structure, extent, and precise location of the plectrum (i.e., the sound-producing area of the forewing), and the structure and function of the resonator^28,29^. Future research should examine how noise exposure affects these traits. Third, although we focused on how noise affects peak frequencies, male grasshoppers may respond to noise by producing louder signals without necessarily elevating frequency. Sound levels are known to be an important trait in mate selection for female crickets^39^, and sound level modification in response to noise has been demonstrated in vertebrates^40,41^. Finally, in addition to male responses examined in this study, it is equally important to assess whether female sound receptor morphology (e.g., tympanic membranes) is affected by noise. This would help determine whether mechanical or neurological filtering reduces the documented impacts of traffic noise on acoustic signaling in Orthoptera.

While previous studies have shown that noise can increase courtship signal frequency across multiple species relying on non-vocal sound production through developmental plasticity and population differentiation^13,22,23^, our study reveals a possible decoupling between morphological and acoustic traits in response to elevated acoustic environments owing to noise pollution. Specifically, although male grasshoppers from noisier roadside habitats exhibited a higher stridulatory file tooth density, a trait often associated with frequency modulation, we found no corresponding increase in signal frequency. This discrepancy suggests species-specific mechanisms in response to acoustic disturbance and highlights the importance of considering multiple traits that interact with acoustic environments to better understand the ecological impacts of noise pollution on individual species and species interactions. Our findings add an important perspective to the growing body of literature on organismal response to the altered acoustic conditions caused by anthropogenic noise.

## Methods

### Insect sampling and environmental measurements

*G. maritimus* is commonly found in sunny and dry grasslands of Japan^42,43^. In addition to these natural habitats, this species is known to occur in anthropogenically disturbed environments such as urban parks and roadside vegetation well-exposed to traffic noise^44^. Our previous research has shown that this species alters its diet composition in response to exposure to traffic noise^45^. Two previous studies on allopatric *C. biguttulus* have also reported that males’ courtship signals in roadside habitats had a higher peak frequency in the 6–9 kHz frequency range than those in non-roadside habitats^13,23^. The frequency peak in low-frequency components in *G. maritimus* can also be spectrally masked by traffic noise (Fig. 1c). We therefore assumed that *G. maritimus* in noisier habitats may represent a morphological change in its sound-producing organs and selected it as a model species in this study.

We established 20 sites across five study regions in Hokkaido, northern Japan (Fig. 1a). The study regions have roadside grasslands exposed to different levels of traffic noise and relatively quiet, natural/semi-natural grasslands. We established 1–2 noisy roadsides and 1–2 quiet sites in each region. For each site, we confirmed that the population density of the focal species was high enough to perform our sampling easily. We collected 7–22 mature males of *G. maritimus* in each site. While sound level measurements would be ideal to perform with a weighing function adjusted for the target species’ hearing range and frequency sensitivity, such information was unavailable. Furthermore, the A-weighted sound pressure level is one of the most widely used metrics in noise management, and an increase in sound pressure level leads to a corresponding increase in the equivalent continuous sound level (Leq), independent of the weighting applied. We therefore quantified 5-min equivalent continuous A-weighted sound pressure level (LAeq) using a sound level meter (TYPE6236, ACO Co., Ltd., Tokyo, Japan) in July-September 2023. We placed the sound level meter at 5 meters from the road edge in roadside sites and otherwise in the center of the site. We repeated these measurements 4–5 times at each site when acoustic disturbance from calling animals and human activities was minimal. We also conducted noise assessments in the summer of 2024 to confirm that noise levels had not changed significantly throughout the survey years (Fig. 1b). All measurements were performed during daytime (9:00–18:00) on sunny and dry weekdays. Some sites became unavailable in 2024 due to a decline in grasshopper populations or the presence of brown bears. In such cases, alternative sites were established (Supplementary Table 1, Supplementary Fig. 1).

We also collected accumulated temperature and precipitation data on each site, as these factors can directly influence the morphological development of grasshoppers or indirectly affect it through the growth of their food plants^46,47^. These were calculated as the sum of average daily temperature or precipitation between May 1 and July 15 (see Supplementary Table 1 for the details of daily temperature and precipitation), corresponding to the average nymphal period of *G. maritimus* predicted by a preliminary investigation. Original data were obtained from the AMeDAS station nearest each study site, distributed on the Japan Meteorological Agency website (https://www.data.jma.go.jp/gmd/risk/obsdl/).

No specific permits were required for the collection of *G. maritimus*, as the species is not regulated under wildlife protection laws in Japan. Ethical approval was not required for this study at Hokkaido University. We have complied with all relevant ethical regulations for animal use.

### Sound recording and analysis

To investigate their acoustic traits, we recorded courtship signals of collected males using a sound recorder (H2N, ZOOM Co., Tokyo, Japan) with a sampling rate of 44.1 kHz, a 16-bit rate, and a WAVE format. We used this sampling rate because a low-frequency peak (<15 kHz) of this species’ courtship signal is expected to be most affected by low-frequency traffic noise^13^ (Fig. 1c). These recordings were conducted on males more than 24 h post-sampling to ensure following a habituation period. However, 21 males were dead before the recordings and were excluded from further procedure. We put a pair of grasshoppers from the same site into the cage to induce the courtship signals. We then recorded 1 and 3–5 courtship signal “phrases”^13^ per a single male in 2023 and 2024, respectively. The distance between a signaling male and the recorder was within the diameter of the cage (i.e., between 1 and 16 cm). After this process, each male was preserved in a 15 ml microtube with 70% ethanol. All recordings were performed in a quiet room with ambient temperature (approx. 20–25 °C).

After the recordings, we checked the sound quality of each recording. Due to low sound quality, we excluded data from 2 individuals and processed data from 276 individuals. For each recording, we trimmed each phrase separately from the recording data using *Audacity version 3.6.2* (*https://www.audacityteam.org/*). We then calculated the peak frequency of each phrase. Peak frequency is an index of the highest relative amplitude of the sound, and is considered an important trait for species and sex recognition in grasshoppers^38^. A previous study on *C. biguttulus* reported that a lower-frequency local maximum in the 6-9 kHz frequency range was affected by traffic noise^13^. On the other hand, our focal species has a lower peak frequency around 10 kHz, and this is out of the frequency band used in the previous study. We therefore did not apply any frequency filters. We performed a 2 kHz high-pass filter to cut off high-amplitude low-frequency noise on each trimmed recording. Acoustic analysis was performed on R version 4.3.2^48^. We used the *readWave* function in *tuneR version 1.4.7*^49^ for reading the sound files and the *meanspec* function in *seewave version 2.2.3*^50^ to measure the peak frequencies. For *meanspec*, we set the parameters of the short-time Fourier transform (STFT) as follows: a window length of 1024 (wl = 1024), 0% overlap (ovlp = 0), and a Hanning window as the window function (wn = “hanning”).

### Morphological measurement

In grasshoppers and crickets, sound-producing organs consist of an exciter (e.g., the number and density of stridulatory file teeth) and a resonator (e.g., specialized regions of the wings). While the morphology and motion of both organs may affect courtship signal frequencies^13,23,51^, we focused on exciter morphology because it is easily quantifiable. Body size is another factor that can affect courtship signal frequencies. We therefore measured exciter morphology and body size of our sampled individuals. For body size measurements, we measured pronotum length to the nearest millimeter as an index of body size, following the above studies (Fig. 2a). For exciter morphology measurements, adult *G. maritimus* males produce acoustic sounds with a plectrum on a forewing and stridulatory files on the inside of a hind leg (Fig. 2b, c). When wings and legs are rubbed together, the plectrum strokes the file teeth, resulting in sound emission^52^. Because the file teeth are distinct, countable organs, we measured the density of file teeth on the legs (i.e., the file length and the number of file teeth) as the focal sound-producing organs of our model species (Fig. 2b, c).

We photographed digital images of the morphological traits of each sample using a stereoscopic microscope (SZX12; Olympus Co., Tokyo, Japan) with a digital camera. Pronotum images were taken from the dorsal side at 7× magnification, while those of the file teeth from the inner side of the hind leg on both sides at 25×. To obtain sharp images for accurate measurements, we generated focus stacking images for each body part using *Photoshop* (Adobe Inc., San Jose, CA, USA) from several pictures taken at different focuses.

Measurements were performed on the images using *ImageJ*^53^. To measure the pronotum length and the file length, we first determined the measurement range (i.e., the range corresponding to the file length) using the “Segmented line” tool, then obtained the number of pixels across the range by the “Measure” tool. This value was finally converted to millimeters divided by conversion rates at each magnification (147.6 pixels/mm at 7× and 539.5 pixels/mm at 25×). These rates were calculated by counting the number of image pixels across 10 divisions of a calibrated eyepiece micrometer photographed at each magnification. We visually counted the number of file teeth using the “Multi-point” tool. File tooth density was finally calculated by dividing the number of file teeth by the file length.

### Statistical analysis

To examine the relationship between sound-producing organs and acoustic environments, we first constructed a generalized linear mixed model (GLMM) with the number of file teeth of each individual as the response variable and the log-transformed file length as an offset term, thereby modeling file tooth density. For this model, we used the combined file tooth number and file length from both hind legs of each individual, since the specific leg (left or right) responsible for generating the acoustic signal corresponding to the peak frequency could not be identified due to technical constraints. Although we selected study sites based on noisy or quiet habitats, road noise conditions in these habitats were relatively variable. Hence, we included the measured LAeq value of each study site in each year as an explanatory variable to capture the effect of variation in noise levels. We also included individual pronotum length, site-specific accumulated temperature and precipitation, and survey year to explain their potential effects on morphological and acoustic traits. We further included interaction terms between survey year and each physiological environmental variable (i.e., LAeq, accumulated temperature, and precipitation) to account for potential year-specific effects. We then fitted two additional GLMMs with file tooth number and file length as response variables to assess their contributions to variation in file tooth density. We used the same set of explanatory variables as in the density model. In addition to these three models, we constructed an additional GLMM to examine the relationship between body size and acoustic and physiological environments. In this model, we used the pronotum length of each individual as the response variable, with the same set of explanatory variables as in the models described above. We included the IDs of study regions and sites as random effects in all four models.

We next analyzed the relationship between courtship signal peak frequency and acoustic environments. We constructed a GLMM including the peak frequency as the response variable. We excluded 7 measurements with peak frequencies exceeding 15,000 Hz from the analysis as potential outliers. We included LAeq, pronotum length, and file tooth density (i.e., file tooth number per file length), survey year, and the interaction term between survey year and LAeq as the explanatory variables. We included the IDs of study regions, sites, and individuals as random effects.

We assumed that all models except for the number of file tooth model follow a normal error distribution with an identity link function. We applied a negative binomial distribution with a log link function for the number of file tooth model because of the likely overdispersion when a Poisson distribution was assumed (dispersion parameter *ϕ* = 2.33). All continuous fixed effects were standardized in model fitting. If the 95% confidence interval for an explanatory variable did not include zero, we interpreted its effect as significant.

### Statistics and reproducibility

All statistical analyses were conducted with *R version 4.3.0*^48^. We used *lmer* or *glmer.nb* functions in *lme4 version 1.1-34*^54^ to build GLMMs, *model_parameters* function in *parameters version 0.24.2*^55^ to calculate the model parameters, and *ggpredict* function in *ggeffects version 1.2.3*^56^ to compute predicted values for the responses. Statistical significance in GLMMs was determined based on whether the 95% confidence intervals of the explanatory variables excluded zero. Replicates were defined at the level of individual grasshoppers for acoustic traits and at the level of sites or regions for morphological data. Non-independence arising from repeated measurements within individuals and clustering within sites and regions was accounted for by including random effects in GLMMs. The model outputs supporting our study can be reproducible in the datasets and code deposited at Figshare^57^ and Zenodo^58^. The details on the exact sample sizes and replicates in each analysis can also be found at Figshare^57^ and Zenodo^58^.

### Reporting summary

Further information on research design is available in the Nature Portfolio Reporting Summary linked to this article.

## Supplementary information

Transparent Peer Review file

Supplementary Information

Reporting Summary

## Data Availability

All raw data, including the numerical source data for the figures, are available in Figshare with the identifier 10.6084/m9.figshare.29244326.

## Code availability

The version of the code used in this study is deposited in Zenodo with the identifier 10.5281/zenodo.19466321. Analyses were performed using R version 4.3.0^48^.
