# Supplementary material for: Male grasshoppers (Glyptobothrus maritimus) in roadside habitats have increased stridulatory sound-producing organs
Source: Commun Biol. 2026 May 5;9:929. doi: 10.1038/s42003-026-10181-4 (PMC13350819; doi:10.1038/s42003-026-10181-4)
Supplement: Supplementary file 2 — Supplementary Information [file 42003_2026_10181_MOESM2_ESM.pdf]

Supplementary Table 1. Detailed coordinates, acoustic environment, and meteorological data at the study sites. The "Survey date" columns show the date on which a grasshopper sampling was conducted. The "AMeDAS" column indicates the name of the observation sites from which the temperatures and precipitations were taken. For details, see the website of the Japan Meteorological Agency (https://www.jma.go.jp/jma/en/Activities/amedas/amedas.html). Hyphens suggest that data were not obtained for the following reasons. In 2024, due to a brown bear presence at TU, OT was established as an alternative. Additionally, grasshopper abundance significantly decreased at TH and KA in 2024. Therefore, we performed sampling at MN as an alternative to TH, but we did not conduct another survey for KA because no suitable sites could be found.

Region	Site	Latitude	Longitude	Acoustic environment	Survey date		LAeq (dBA)		AMeDAS	Temperature		Precipitation	
					2023	2024	2023	2024		2023	2024	2023	2024
AS	KP	43.747	142.314	Quiet	2023/9/8	2024/7/18	42.5	40.4	Asahikawa	1300.7	1279	236.5	158
AS	MC	43.690	142.087	Quiet	2023/9/8	2024/7/18	45	40.7	Fukagawa	1240.3	1231	138.5	165
AS	OA	43.709	142.144	Noisy	2023/9/8	2024/7/18	67.4	60	Fukagawa	1240.3	1231	138.5	165
HD	FC	41.768	140.872	Quiet	2023/8/23	2024/7/31	56.5	40.1	Hakodate	1301	1308.4	264	206
HD	KK	41.844	140.740	Noisy	2023/8/23	2024/7/31	69	70.6	Hakodate	1301	1308.4	264	206
HD	NE	41.820	140.693	Noisy	2023/8/23	2024/7/31	67.5	65.2	Hakodate	1301	1308.4	264	206
KS	MP	43.069	144.466	Quiet	2023/8/14	2024/8/10	34	37.9	Kushiro	1054.9	1032.5	224.5	145
KS	TU	43.114	144.314	Quiet	2023/8/14	-	33.3	-	Kushiro	1054.9	-	224.5	-
KS	KT	43.001	144.237	Noisy	2023/8/14	2024/8/10	68.4	66.7	Kushiro	1054.9	1032.5	224.5	145
KS	KC	43.002	144.419	Noisy	2023/8/14	2024/8/10	65.5	65.6	Kushiro	1054.9	1032.5	224.5	145
KS	OT	43.014	144.292	Quiet	-	2024/8/10	-	41.3	Kushiro	-	1032.5	-	145
SP	HU	43.083	141.337	Quiet	2023/8/10	2024/7/22	52	50.4	Sapporo	1340.1	1335.4	206	85.5
SP	TH	43.073	141.404	Quiet	2023/8/10	2024/8/6	45.6	43.3	Sapporo	1340.1	1335.4	206	85.5

SP	NT	43.101	141.344	Noisy	2023/8/10	2024/7/22	71	70.5	Sapporo	1340.1	1335.4	206	85.5
SP	JT	43.079	141.419	Noisy	2023/8/10	2024/8/6	72.5	73.4	Sapporo	1340.1	1335.4	206	85.5
TK	CF	42.932	143.240	Quiet	2023/8/15	2024/8/15	39.3	32.8	Obihiro	1273.8	1267.8	156.5	106.5
TK	HP	43.129	143.627	Quiet	2023/8/15	2024/8/15	35.6	44	Honbetsu	1231.8	1216.3	108	88
TK	TR	42.940	143.202	Noisy	2023/8/15	2024/8/15	62.8	68.8	Obihiro	1273.8	1267.8	156.5	106.5
TK	KA	43.115	143.638	Noisy	2023/8/15	-	64.4	-	Honbetsu	1231.8	-	108	-

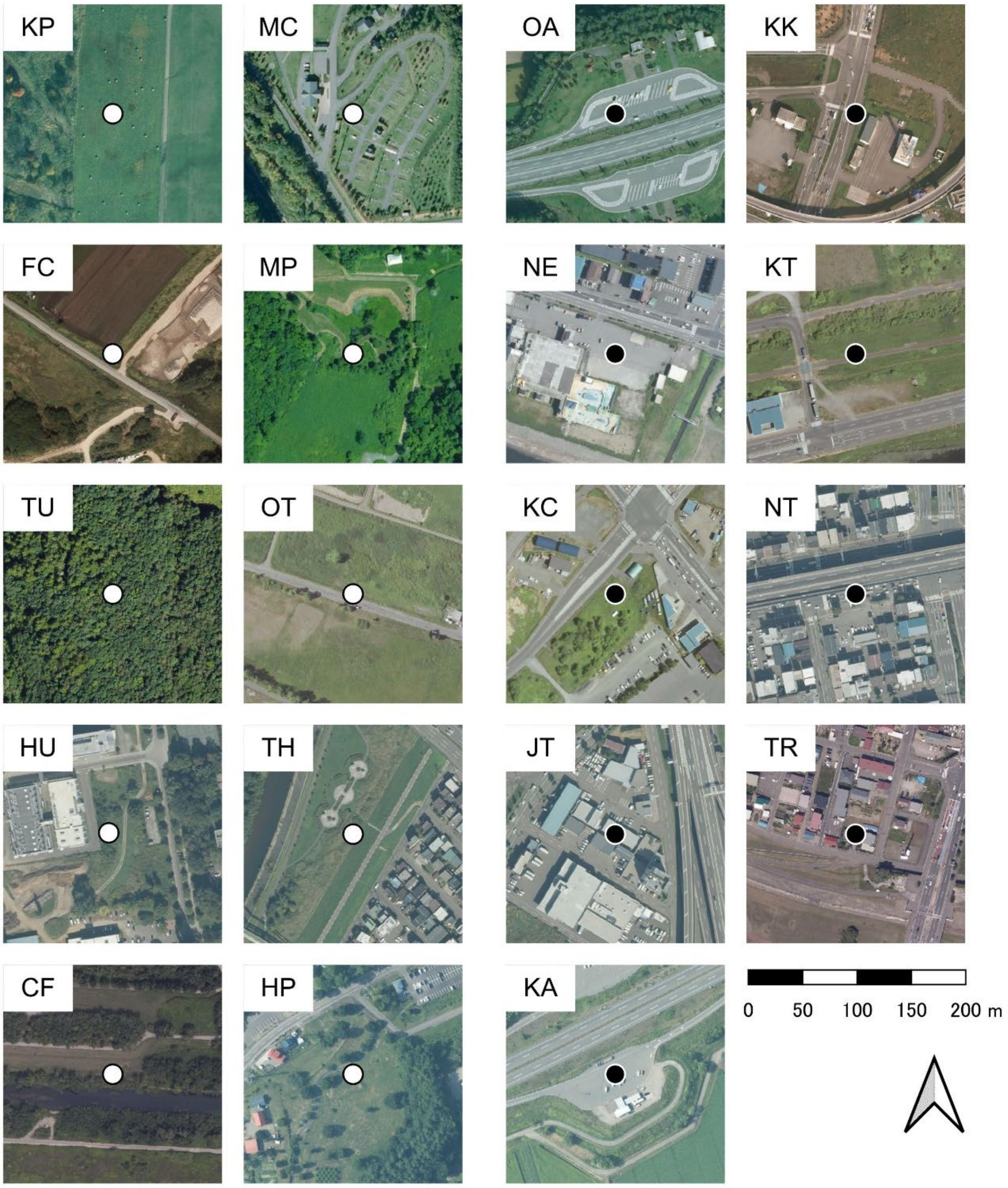

Supplementary Figure 1. The landscape around each study site. Sites with a quiet acoustic environment are indicated by white circles, while noisy sites are indicated by black circles. Seamless aerial photographs published by the Geospatial Information Authority of Japan (GSI) were used as the base map.
