# Supplementary material for: Male grasshoppers (Glyptobothrus maritimus) in roadside habitats have increased stridulatory sound-producing organs
Source: Commun Biol. 2026 May 5;9:929. doi: 10.1038/s42003-026-10181-4 (PMC13350819; doi:10.1038/s42003-026-10181-4)
Supplement: Supplementary file 3 — Reporting Summary [file 42003_2026_10181_MOESM3_ESM.pdf]

nature portfolio

Corresponding author(s): *Double-anonymous peer review submissions: write DAPR and your manuscript number here instead of author names.*

Last updated by author(s): *YYYY-MM-DD*

Reporting Summary

Nature Portfolio wishes to improve the reproducibility of the work that we publish. This form provides structure for consistency and transparency in reporting. For further information on Nature Portfolio policies, see our Editorial Policies and the Editorial Policy Checklist.

Statistics

For all statistical analyses, confirm that the following items are present in the figure legend, table legend, main text, or Methods section.

n/a	Confirmed	
☐	☒	The exact sample size (*n*) for each experimental group/condition, given as a discrete number and unit of measurement
☐	☒	A statement on whether measurements were taken from distinct samples or whether the same sample was measured repeatedly
☐	☒	The statistical test(s) used AND whether they are one- or two-sided<br>*Only common tests should be described solely by name; describe more complex techniques in the Methods section.*
☐	☒	A description of all covariates tested
☐	☒	A description of any assumptions or corrections, such as tests of normality and adjustment for multiple comparisons
☐	☒	A full description of the statistical parameters including central tendency (e.g. means) or other basic estimates (e.g. regression coefficient) AND variation (e.g. standard deviation) or associated estimates of uncertainty (e.g. confidence intervals)
☐	☒	For null hypothesis testing, the test statistic (e.g. *F*, *t*, *r*) with confidence intervals, effect sizes, degrees of freedom and *P* value noted<br>*Give P values as exact values whenever suitable.*
☒	☐	For Bayesian analysis, information on the choice of priors and Markov chain Monte Carlo settings
☐	☒	For hierarchical and complex designs, identification of the appropriate level for tests and full reporting of outcomes
☐	☒	Estimates of effect sizes (e.g. Cohen's *d*, Pearson's *r*), indicating how they were calculated

Our web collection on statistics for biologists contains articles on many of the points above.

Software and code

Policy information about availability of computer code

Data collection	Morphological measurements of grasshoppers on photographs were performed using ImageJ. We visually counted the number of sound-producing organs using the “Multi-point” tool. Acoustic data were collected from each grasshopper sample in a laboratory setting with a standard condition. We have measured the sound pressure levels of traffic noise in each study site. We have obtained climate data of each study site from the Japan Meteorological Agency website (https://www.data.jma.go.jp/gmd/risk/obsdl/), which is available for free. We have uploaded all the code and data in Figshare: 10.6084/m9.figshare.29244326.
Data analysis	All statistical analyses of this manuscript were conducted with version 4.3.0. We have uploaded all the code and data in Figshare: 10.6084/m9.figshare.29244326.

For manuscripts utilizing custom algorithms or software that are central to the research but not yet described in published literature, software must be made available to editors and reviewers. We strongly encourage code deposition in a community repository (e.g. GitHub). See the Nature Portfolio guidelines for submitting code & software for further information.

Data

Policy information about availability of data

All manuscripts must include a data availability statement. This statement should provide the following information, where applicable:

- Accession codes, unique identifiers, or web links for publicly available datasets
- A description of any restrictions on data availability
- For clinical datasets or third party data, please ensure that the statement adheres to our policy

We have collected the original data for this research. The data and code are available in Figshare: 10.6084/m9.figshare.29244326.

Research involving human participants, their data, or biological material

Policy information about studies with human participants or human data. See also policy information about sex, gender (identity/presentation), and sexual orientation and race, ethnicity and racism.

Reporting on sex and gender	No human participants have been involved to this research and we have not used any Human research materials.
Reporting on race, ethnicity, or other socially relevant groupings	No human participants have been involved to this research and we have not used any Human research materials.
Population characteristics	No human participants have been involved to this research and we have not used any Human research materials.
Recruitment	No human participants have been involved to this research and we have not used any Human research materials.
Ethics oversight	No human participants have been involved to this research and we have not used any Human research materials.

Note that full information on the approval of the study protocol must also be provided in the manuscript.

Field-specific reporting

Please select the one below that is the best fit for your research. If you are not sure, read the appropriate sections before making your selection.

☐ Life sciences ☐ Behavioural & social sciences ☒ Ecological, evolutionary & environmental sciences

For a reference copy of the document with all sections, see nature.com/documents/nr-reporting-summary-flat.pdf

Ecological, evolutionary & environmental sciences study design

All studies must disclose on these points even when the disclosure is negative.

Study description	We established five study regions in Hokkaido, northern Japan, for insect sampling. We then established a maximum of two noisy-roadside and two quiet sites in each study region, resulting in 20 study sites (10 noisy-roadside and 10 quiet sites). The sound pressure level values (i.e., LAeq values, mean ± standard deviation) were 67.6±3.51 dBA in noisy sites and 41.9±6.45 dBA in quiet sites, respectively. We collected 7–22 males of Glyptobothrus maritimus at each site during the summers of 2023 and 2024. We also obtained the average daily temperature and precipitation between May 1 and July 15 from a free dataset distributed by the Japan Meteorological Agency website (https://www.data.jma.go.jp/gmd/risk/obsdl/). For each sampled male grasshopper, we recorded 1~5 courtship signals in a laboratory setting and then preserved them in a 15 ml micro-tube with 70% ethanol. We finally measured body size and sound-producing organs (i.e., the number and density of stridulatory file teeth) of each individual photographed with a stereoscopic microscope. For these measurements, we used the ImageJ software.
Research sample	We collected a total of 299 intact male G. maritimus individuals from both noisy-roadside and quiet habitats, across the five regions. We obtained the data of body size, the number of sound-producing organs, and peak frequency of each male individual.
Sampling strategy	To examine the difference in the sound-producing organs and peak frequency with acoustic conditions, we adopted a large-scale nested sampling design; we established multiple noisy-roadside and quiet habitats in each of five study regions. We also repeated sampling across two summer seasons. The spatial-temporal scale of this design is large enough compared to that of previous studies.
Data collection	For the peak frequency analysis, we trimmed each phrase separately from the recording data using Audacity version 3.6.230. We then automatically obtained the peak frequency using a custom code in R. We repeated these procedures twice with different authors (JY and KN) and confirmed that we could reproduce the values. For morphological measurement, we photographed digital images of the morphological traits of each sample using a stereoscopic microscope (SZX12; Olympus Co., Tokyo, Japan) with a digital camera. Pronotum images were taken from the dorsal side at 7× magnification, while those of the file teeth from the inner side of the hind leg on both sides at 25×. To obtain sharp images for accurate measurements, we generated focus stacking images for each body part using Photoshop (Adobe Inc., San Jose, CA, USA) from several pictures taken at different focuses. We conducted these with three different authors (JY, KN, and TH). For both analysis and measurement, we labeled each recording and sample with the

	information of the sampling year, date, site, and region so that we strictly managed to avoid double sampling from the same individual.
Timing and spatial scale	We conducted our insect sampling during the peak period of breeding/reproductive season (mid July to early September) of our target species in 2023 and 2024. We established study sites in multiple landscapes in Hokkaido, northern Japan, covering approximately 75000 km^2, and obtained acoustic and morphological data from 7–22 collected male grasshoppers in each site.
Data exclusions	We did not sample data of injured individuals.
Reproducibility	We double-checked the reproducibility of acoustic analysis and morphological measurement with multiple authors, and all attempts to repeat the acoustic analysis and morphological measurement were successful. We also have uploaded all the code and data in Figshare: 10.6084/m9.figshare.29244326, and confirmed any statistical values have been reproduced.
Randomization	We examined the effect of the acoustic condition (noisy or quiet) on grasshopper sounds and morphology, and we were unable to assign the treatment of the acoustic condition randomly because we conducted our sampling in noisy-roadside and quiet sites. We therefore accounted for any covariates (i.e., average temperature and precipitation of each site and temperature and humidity of lab recording) that can potentially affect grasshopper sounds and morphology in the statistical analysis.
Blinding	To avoid any unconscious bias, we blinded the information about the acoustic conditions of the sampling site for the morphological measurement.

Did the study involve field work? ☒ Yes ☐ No

Field work, collection and transport

Field conditions	We conducted our insect sampling during the peak period of breeding/reproductive season (mid July to early September) of our target species in 2023 and 2024. The sum of average daily temperature and precipitation of the study sites between May 1 and July 15 was 1033-1340 degrees and 88-225 mm, respectively.
Location	Hokkaido, northern Japan (41°33'N to 45°33'N, 139°20'E to 146°13'E).
Access & import/export	All data were obtained in accordance with the current laws of Japan and with relevant international guidelines and regulations.
Disturbance	We tried to sample a minimum number of insects at each site. The target species were abundant, and therefore we assumed that our sampling had a minimum ecological impact.

Reporting for specific materials, systems and methods

We require information from authors about some types of materials, experimental systems and methods used in many studies. Here, indicate whether each material, system or method listed is relevant to your study. If you are not sure if a list item applies to your research, read the appropriate section before selecting a response.

Materials & experimental systems

n/a	Involved in the study	
☒	☐	Antibodies
☒	☐	Eukaryotic cell lines
☒	☐	Palaeontology and archaeology
☐	☒	Animals and other organisms
☒	☐	Clinical data
☒	☐	Dual use research of concern
☒	☐	Plants

Methods

n/a	Involved in the study	
☒	☐	ChIP-seq
☒	☐	Flow cytometry
☒	☐	MRI-based neuroimaging

Animals and other research organisms

Policy information about studies involving animals; ARRIVE guidelines recommended for reporting animal research, and Sex and Gender in Research

Laboratory animals	We have not used any laboratory animals.
Wild animals	We focused on G. maritimus and collected adult male this species in the field sites using a insect net. We preserved all samples in a 15 ml micro-tube with 70% ethanol.
Reporting on sex	Not applicable.

Field-collected samples	All samples after the measurement have been stored at the EES building of Hokkaido University.
Ethics oversight	No ethical approval was needed because the study was conducted on an invertebrate species.

Note that full information on the approval of the study protocol must also be provided in the manuscript.

Plants

Seed stocks	We did not collect the plant seeds.
Novel plant genotypes	We did not collect the plant genes.
Authentication	We did not collect the plants.
