# Supplementary material for: Male grasshoppers (Glyptobothrus maritimus) in roadside habitats have increased stridulatory sound-producing organs
Source: Commun Biol. 2026 May 5;9:929. doi: 10.1038/s42003-026-10181-4 (PMC13350819; doi:10.1038/s42003-026-10181-4)
Supplement: Supplementary file 1 — Transparent Peer Review file [file 42003_2026_10181_MOESM1_ESM.pdf]

nature portfolio

Peer Review File

Male grasshoppers (Glyptobothrus maritimus) in roadside habitats have increased stridulatory sound-producing organs

Corresponding Author: Dr Masayuki Senzaki

This file contains all reviewer reports in order by version, followed by all author rebuttals in order by version.

Attachments originally included by the reviewers as part of their assessment can be found at the end of this file.

Version 0:

Reviewer comments:

Reviewer #1

(Remarks to the Author)
This paper makes a fresh and important contribution by showing what could be the first signs of morphological adaptation to human-made noise in a non-vocal insect. By examining grasshopper sound-producing organs across different habitats, the study broadens our view of how noise can affect wildlife well beyond the usual focus on vocal species. The multi-site approach adds weight to the findings, and the ecological angle is genuinely interesting.
Still, a few big questions keep the conclusions from feeling fully secure. The study doesn't yet nail down whether the observed physical changes actually affect signals, especially since factors like wing movement speed could also be in play. Also, the recordings under a noisy environment are key to establish some of the speculations. Some choices in the analysis and data presentation, like the filter selection, how 'year' was handled, and the way results were shown, could use tightening to back up the claims.
In short, it's an exciting and thought-provoking piece of work that opens up new avenues, but it would be even stronger with some other data, clearer methods and stronger evidence linking the morphological changes to real communication benefits.

Specific comments
Line 78: Were there any calls happening during the LAeq measurement? I am curious to know the context.
Line 84: Wing speed can also cause an increase in frequency. It is also important to examine the same.
Line 107: What is the call frequency range of the grasshoppers? Also, any information about the female hearing? As those are important to determine the filter to use, as A filter is mostly for the human auditory range.
Line 122: A mean and SD is a better way of representing the data.
Line 134: recorded 1 and 3~5 courtship signal ??
Line 220: Did the authors check all the model fits, using standard plotting?
Result section: I don't see a table for the random effects, i.e. the year. Also, if we consider this selection pressure to be ongoing across the years, I would rather consider years as a fixed effect, as the 2024 population is a byproduct of the 2023 population that survived under selection.
Line 252: To maintain parity and ease of understanding, please plot file density against pronotum length (Fig. 4).
Line 272: Exactly, this is a key point which needs to be tested. Also, are the wing movement velocities identical between the populations?
Line 311: A bit hand-wavy unless tested.

Reviewer #2

(Remarks to the Author)
Review of the manuscript entitled "Male grasshoppers in roadside habitats have increased stridulatory sound-producing organs" by Jou Yoshigai, Keisuke Nakaoka, Tomoki Hirose, Ryugo Ishida, Daiki Waga, Takehiko Shimizu and Masayuki Senzaki.
Anthropogenic noise threatens the integrity of acoustic communication, and the manuscript examines whether grasshoppers adjust stridulatory morphology and signal frequency in noisy roadside habitats. The study found that male grasshoppers

(Glyptobothrus maritimus) from noisy roadside habitats had approximately 3.5% more stridulatory file teeth per unit length compared to those from quiet habitats, providing evidence of morphological adaptation to anthropogenic noise in non-vocal animals. However, despite this morphological change, there was no significant increase in courtship signal frequency, suggesting a decoupling between morphological and acoustic traits. Interestingly, this finding contrasts with previous studies on related species, highlighting that morphological adaptations to noise may not always translate into functional acoustic benefits.

Although the dataset is valuable and the core questions are timely, several aspects of the statistical workflow, figure design, and inferential logic can be improved or clarified:

1. The authors report 306 total individuals (152 noisy and 154 quiet), but the actual dataset contains only 299 individuals (149 noisy and 150 quiet). Did I miss something? Could the authors clarify?

2. Seven measurements exceed 15,000 Hz (normal range ~6,000-12,000 Hz). Could they be measurement errors or outliers? If removed, does the trend hold?

3. Although the binary design (Quiet vs. Noisy) is a pragmatic solution, it oversimplifies the actual spectrum of acoustic disturbances in nature. I would strongly recommend including at least one intermediate step – a moderate level - and reanalysing the data. Reclassifying into three ecologically distinct categories may provide clearer biological insights and confirm that the main findings (e.g., morphological changes) persist and are enhanced in interpretability when accounting for moderate noise exposure zones. After inspecting the dataset, I am confident that it has potential. For example - Quiet: ≤ 48 dBA; Moderate: 48 < LAeq < 60 dBA; Noisy: ≥ 60 dBA.

4. The authors generated focus stacking images for each body part using Photoshop extensively. Using Photoshop for focus stacking and image analysis could introduce several biases and measurement errors that might affect the reliability of the morphological findings. As far as I know, Photoshop is not considered optimal for focus stacking compared to specialised software (e.g., https://community.adobe.com/t5/photoshop-ecosystem-discussions/focus-stacking-in-photoshop-horrible-results/m-p/8960498). Since automated systems might show some measurement variation compared to manual methods, and the study's key finding is a 3.5% difference, focus stacking, hence, artifacts could potentially account for a substantial portion of the observed difference. To assess whether Photoshop introduced significant bias, I recommend:
- Repeat measurements on a subset using specialised focus stacking software (e.g., zero-cost open-source tools: ImageJ EDF, CombineZP, PICOLAY, etc.; or non-free Helicon Focus, Zerene Stacker; however, Zerene must have a free trial period, if I am not mistaken)
- Compare with direct ImageJ analysis of the same specimens
- Use the raw images and validate the results, if possible

5. How meaningful is it to include the year (only two levels) in the models as random factors? To show the temporal consistency between the years, wouldn't it be more meaningful to include the year as fixed effects? It still statistically works this way since not all sites in 2023 were resampled in 2024, and thus there is no perfect overlap between the sites and year factors.

6. Twenty or more individuals in the morphology dataset lack peak frequency measurements. I might have missed that, but could the authors explain?

7. The authors repeatedly describe the morphological changes as "adaptive responses" without providing sufficient evidence. The authors make strong evolutionary claims based on correlative data. The 3.5% difference in file teeth density could equally result from:
- Developmental plasticity responses to stress
- Population genetic drift in isolated roadside populations
- Indirect selection on correlated traits
- Environmental effects on resource allocation during development
Consider using more cautious language and explicitly discuss alternative hypotheses.

8. The discussion fails to adequately explore that the morphological changes might be maladaptive byproducts of developmental stress. The authors briefly mention this possibility but don't fully develop the argument that increased file teeth without frequency elevation could impose energetic costs without communication benefits. Consider expanding/adding a paragraph in the discussion section.

9. I think the discussion section is narrowly based on two studies of Chorthippus biguttulus. The authors should incorporate the broader literature. For example, focusing on:
- Stress-induced morphological variation in also other insect species
- Developmental constraints on acoustic organ evolution
- Trade-offs between signal production and other fitness components
- Population genetic studies showing neutral evolution of acoustic traits

10. The authors compare Glyptobothrus maritimus with C. biguttulus, focusing primarily on baseline frequencies (10 kHz vs 6-9 kHz), but ignore possible:
- Phylogenetic relationships and evolutionary constraints
- Ecological differences beyond noise exposure

- Methodological differences between studies
- Sample size and statistical power comparisons

11. Unfortunately, the discussion also focuses on oversimplified views of grasshopper acoustic evolution, ignoring recent research on complex genetic architectures underlying acoustic traits and multiple developmental pathways affecting stridulatory morphology, as well as possibly frequency-independent functions of file tooth variation and context-dependent expression of acoustic phenotypes.

12. The figures look inconsistent. In Fig. 3, the predicted relationship between pronotum length (x) and peak frequency (y) is plotted, excluding the interaction pronotum x environment. In Fig. 4, however, the interaction is included due to significance. For Fig. 3, consider adding separate-coloured lines (as in Fig. 4) to make the grouping variable visually explicit. Also, plot simple box/violin plots of peak frequency by habitat – I believe this direct visual group comparison is missing and will be expected by most readers.

13. I might have missed that, but did the authors investigate possible fluctuating asymmetry (FA)? Statistically, the trait differences the study focuses on (file-tooth density) are therefore unaffected by left-right effects. Consequently, omitting an explicit FA analysis did not bias the main conclusions; however, reporting it would have strengthened the methodological transparency and the discussion section.

14. Consider also reporting P values along with the 90% and 95% confidence intervals. I am personally not a big fan of P values either. However, most readers might still find them intuitive, and I believe it is always better to report more than less.

Minor issues:

15. L24 – Authors mentioned that grasshoppers from noisy sites had a greater number of sound-producing organs… Please note also greater than what, and what is the magnitude (in times or percentages). Please also add values (e.g., percentages) wherever possible. Simply stating “higher” or “lower” is not descriptive (e.g., L114-115).

16. L42: Replace “is” with “are”

17. L229-230: Consider moving this info to the methods section

18. L231-232: Consider creating a small table here with reported values

19. Consider adding the species name as a keyword, as it is not mentioned in the title.

20. Fig. 2: “G. maritimus” should be in italic

Reviewer #3

(Remarks to the Author)
Manuscript title: Male grasshoppers in roadside habitats have increased stridulatory sound-producing organs.
This research investigates the morphological adaptations in the sound-producing organs of male grasshoppers, specifically in response to anthropogenic noise. The study highlights the potential changes in their courtship signals, emphasising the significance of understanding how environmental factors can influence these vital communication mechanisms, specifically changes in the stridulatory apparatus.

The paper explores a significant topic within the field of ecoacoustics, specifically examining how animal communities respond to anthropogenic activities. Although the impacts of urban noise on bird and bat populations have been extensively studied, research on insects remains limited. Therefore, this study offers insights into one possible adaptation to anthropogenic noise. Nevertheless, the findings do not suggest any changes in sound production structures or the peak frequency of acoustic signals related to anatomical modifications. As such, the authors recommended that future research should focus on behavioural traits.
The findings presented in the manuscript provide a novel perspective; however, the manuscript is not conclusive and fails to cite pioneering work on grasshopper stridulation to show additional knowledge of the mechanism (see recommended references). The study could also be enhanced by incorporating additional factors. For example investigating behavioural aspects that influence the localisation of the singing individual (such as ground level, grass, cavities, etc.) to improve signal transmission, time of peak activity, or examining social interactions that affect the distribution of males and females would be beneficial. Additionally, a thorough analysis of other acoustic properties besides peak frequency—such as sound pressure levels, calling rates, and song patterns—would further enrich the understanding of the effects of noise on grasshopper communities.

Specific comments:
1) Road noise is an environmental factor that could influence acoustic communication among grasshoppers. However, the primary focus of the research was on morphological modifications. In our opinion, if road noise does affect the acoustic communication in the examined grasshopper species, one might anticipate initial behavioural adaptations before any noticeable morphological changes occur. Behavioural modifications represent a more flexible response compared to morphological changes. Among these adjustments, grasshoppers may choose to sing from specific locations that enhance sound propagation, such as at ground level, or they may alter their population distribution, resulting in individuals being

closer to one another. Did the authors gather descriptive information about the studied populations, such as the number of individuals, the location of singing males, and records of social interactions?

2) Conversely, the assessment of morphological changes did not consider the hearing organs, particularly the tympanic membranes in females, which serve as the primary receivers of acoustic signals. It is also possible that road noise does not significantly impact the hearing process. Therefore, it would be essential to investigate whether there is any mechanical or neurological filtering mechanism in place that renders road noise irrelevant as an environmental pressure driving adaptive changes. If females were collected, can their tympanic membranes be measured? Additionally, are there records of female responses to noise?

3) It appears that researchers have gathered valuable data from the communities under study. I would suggest that the authors examine the community structure and the dynamics of changes that occurred throughout the study. This exploration could provide further insights into how road noise affects grasshopper populations, enhancing the understanding of their behaviour and interactions in response to environmental disturbances.

Lines 53-53: by stridulating sound-producing organs at relatively constant speeds….
How do you know this? if you see classical papers of von Helversen & Helversen (J Comp Physiol A (1997) 180: 373-386), (Tarasova et al., 2021) The wing motion patterns suggest variable speeds during stridulation. We noticed that you cite Helversen & Helversen (1997), but you did not use it to analyse the stridulatory motion patterns, which suggests variable speeds.
Line 56-57. You can still have such plasticity without noticeable morphological changes, for example, if the speed increases, causing a high rate of tooth impacts on the stridulatory files.
Lines 76-84: all of this here should be placed in methods.
Lines 91-95. Do both species live in sympatry?
Lines 113-117: Even if you are trying to demonstrate that the two sites differ in background noise levels, this should be placed in the results.
Line 131: I'm very sorry, but this sampling rate is too low; you might be underestimating frequencies > 20 kHz. If you see the paper by Lampe et al, the sampling frequency used in their recordings was 192 kHz, especially because the calling song of this species has significant energy between 7 and 45 kHz.
Lines 133-135: more details here, what was the distance between microphone and the singing insect? did you measure sound levels of the recorded songs? why not?
Line 143: "peak frequencies of C. biguttulus in the 6-9 kHz frequency…" This is only the dominant frequency, but you are missing a huge amplitude component at about 37 kHz.
Lines 156-157: "Body size and sound-producing organs, such as the number and density of stridulatory file teeth…" What about radiator's resonances?
Lines 164-166: Yes, but crickets use a different mechanism; they need control of the carrier frequency to maintain a single tone, and use an escapement mechanism which depends on systemically distributed teeth (among other factors), which will not apply to the grasshoppers as they use broadband signals.
Lines 230-233: This here is confusing. Can you present the stats in a more organised way?
Figures and table: can you please present the equation parameters: slope, intercepts and other relevant statistics?
Lines 239-242: The length of the file in relation to body size has been shown in other stridulating orthoptera, crickets and katydids (Chivers et al., 2017; Montealegre-Z et al., 2017), so it is not surprising that the extra number of teeth correlates with body size. And in relation to this, a coherent expectation would be that longer files are associated with lower frequencies.
Discussion
Lines 256-259: Following from my comments above, relating to body size, file length, etc, this section deserves a better interpretation. For example, you should focus more on exploring sound levels, under the hypothesis that those insects living in regions exposed to human noise tend to sing louder. For this you will need properly calibrated equipment. In crickets for example, large males tend to be louder (Gray, 1997).
In summary, the authors should provide additional interpretation of the data to produce a more coherent manuscript.

Recommended references
Chivers, B. D., Jonsson, T., Soulsbury, C. D. & Montealegre-Z, F. 2017. Structural biomechanics determine spectral purity of bush-cricket calls. Biol. Lett. 13.

Gray, D. A. 1997. Female house crickets,Acheta domesticus, prefer the chirps of large males. Animal Behaviour 54: 1553-1562.

Montealegre-Z, F., Ogden, J., Jonsson, T. & Soulsbury, C. D. 2017. Morphological determinants of signal carrier frequency in katydids (Orthoptera): a comparative analysis using biophysical evidence of wing vibration. J. Evol. Biol. 30: 2068-2078.

Tarasova, T. A., Sevastianov, N. S. & Vedenina, V. Y. 2021. Songs and morphology in grasshoppers of the Stenobothrus eurasius group (Orthoptera: Acrdidae: Gomphocerinae) from Russia and adjacent countries: clarifying of taxonomic status. Zootaxa 4965: 244260.

Reviewer #4

(Remarks to the Author)
I co-reviewed this manuscript with one of the reviewers who provided the listed reports. This is part of the Communications Biology initiative to facilitate training in peer review and to provide appropriate recognition for Early Career Researchers who

co-review manuscripts.

Version 1:

Reviewer comments:

Reviewer #1

(Remarks to the Author)
No further comments. Please accept as is. I am happy with the responses.

Reviewer #2

(Remarks to the Author)
I am very pleased to see that the authors have incorporated and accepted almost all of my previous comments, and I am very grateful for their efforts. I also understand why some of my suggestions were not implemented, as the authors provided clear and convincing arguments in those cases. I would be happy to see this manuscript published in its current form.

Reviewer #3

(Remarks to the Author)
No comments

Reviewer #4

(Remarks to the Author)
I co-reviewed this manuscript with one of the reviewers who provided the listed reports. This is part of the Communications Biology initiative to facilitate training in peer review and to provide appropriate recognition for Early Career Researchers who co-review manuscripts.

Open Access This Peer Review File is licensed under a Creative Commons Attribution 4.0 International License, which permits use, sharing, adaptation, distribution and reproduction in any medium or format, as long as you give appropriate credit to the original author(s) and the source, provide a link to the Creative Commons license, and indicate if changes were made.
In cases where reviewers are anonymous, credit should be given to 'Anonymous Referee' and the source.
The images or other third party material in this Peer Review File are included in the article’s Creative Commons license, unless indicated otherwise in a credit line to the material. If material is not included in the article’s Creative Commons license and your intended use is not permitted by statutory regulation or exceeds the permitted use, you will need to obtain permission directly from the copyright holder.
To view a copy of this license, visit https://creativecommons.org/licenses/by/4.0/

#Reviewers' comments

Reviewer #1 (Remarks to the Author):

##### Comment

This paper makes a fresh and important contribution by showing what could be the first signs of morphological adaptation to human-made noise in a non-vocal insect. By examining grasshopper sound-producing organs across different habitats, the study broadens our view of how noise can affect wildlife well beyond the usual focus on vocal species. The multi-site approach adds weight to the findings, and the ecological angle is genuinely interesting.

Still, a few big questions keep the conclusions from feeling fully secure. The study doesn't yet nail down whether the observed physical changes actually affect signals, especially since factors like wing movement speed could also be in play. Also, the recordings under a noisy environment are key to establish some of the speculations. Some choices in the analysis and data presentation, like the filter selection, how 'year' was handled, and the way results were shown, could use tightening to back up the claims.

In short, it's an exciting and thought-provoking piece of work that opens up new avenues, but it would be even stronger with some other data, clearer methods and stronger evidence linking the morphological changes to real communication benefits.

Response: Thank you very much for your positive evaluation of our research with fair and critically important views. We have tried to address all your concerns to make it stronger. See below for our specific revisions.

##### Comment

Specific comments

Line 78: Were there any calls happening during the LAeq measurement? I am curious to know the context.

Response: We have tried to conduct these measurements when no acoustic disturbance, including insect sounds, occurs. We have added this in the Method section.

##### Comment

Line 84: Wing speed can also cause an increase in frequency. It is also important to examine the same.

Response: Thank you very much for sharing this critical view in explaining higher frequency sounds in grasshoppers in the literature. Although we agree with this,

measuring this behavior is especially important for roadside populations that produce higher frequency sounds under either noise or ambient conditions. The higher frequency in noise may be possible for the studied populations; however, we have no such evidence. We have therefore discussed this possibility in the 5th and 7th paragraphs of the discussion.

Comment

Line 107: What is the call frequency range of the grasshoppers? Also, any information about the female hearing? As those are important to determine the filter to use, as A filter is mostly for the human auditory range.

Response: We are grateful for pointing this out. To clarify the spectral overlap in peak frequency between traffic noise and the courtship signal of the target species, we have added those figures in Figure 1c, and added a sentence indicating potential spectral masking of traffic noise on courtship signals in G. maritimus. We also do not know the female hearing of this species. Because we agree with this point, we have added the need for research on female responses in future work in the discussion section. As for the A filter, we have used it as this filter provides useful management information, and Leq values will increase regardless of the weighting choices in the SPL increases. Ideally, a weighing function adjusted for grasshopper hearing ability is needed, but this is unavailable. We have clarified these in the method section.

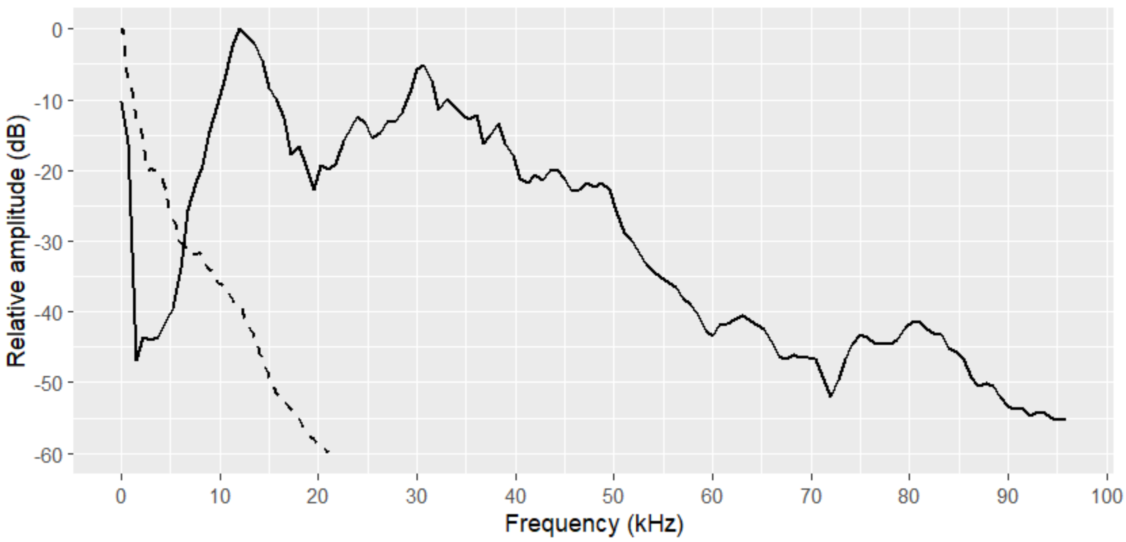

Figure. Power spectra of the grasshopper *G. maritimus* courtship signal (solid line) and road noise (broken line). The horizontal axis represents sound frequencies, and the vertical axis shows the relative amplitude of each frequency. The grasshopper signal

was recorded using a microphone Ultramic 384K EVO (DODOTORONIC, RM, Italy) in a soundproof room. The road noise was obtained at the noisy site NT in the region SP (please see the supplement).

Comment

Line 122: A mean and SD is a better way of representing the data.
Response: We have added the mean and SD data to the supplementary table.

Comment

Line 134: recorded 1 and 3~5 courtship signal ??
Response: Thank you very much for this. The complete sentence here is "We then recorded 1 and 3~5 courtship signal "phrases" per a single male in 2023 and 2024, respectively."

Comment

Line 220: Did the authors check all the model fits, using standard plotting?
Response: We appreciate the reviewer's feedback for informing us of the model diagnostics, in which we fully agree with the importance of providing such information. For all models, although those are reanalyzed models based on reviewers' feedback, we have checked variance inflation factors, determination coefficients (marginal and conditional R^2), QQ-plots with tests of normality, overdispersion, and outliers, and have confirmed that all of these are within a good fit. We have included those results (see below) in Table 1.

Response variable	conditional R^2	marginal R^2	Normality(KS test)	Overdispersion	Outlier
Peak frequency	0.37	0.07	p = 0.06	p = 0.73	p = 0.49
Pronotum length	0.39	0.04	p = 0.06	p = 0.6	p = 1
File length	0.56	0.33	p = 0.46	p = 0.49	p = 1
Number of file teeth	0.30	0.23	p = 0.68	p = 0.85	p = 0.96
Density of file teeth	0.57	0.28	p = 0.56	p = 0.59	p = 0.98

Comment
Result section: I don't see a table for the random effects, i.e. the year. Also, if we consider this selection pressure to be ongoing across the years, I would rather consider years as a fixed effect, as the 2024 population is a byproduct of the 2023 population that survived under selection.
Response: Thank you for your suggestion. Firstly, we have added standard deviations of random effects to the table of results (Table 1). Second, as you suggested, we have included "Year" as fixed effects rather than random effects to directly account for the yearly variations of the response variables.

Regarding this modification, we additionally included interaction terms between year and other traits and environmental variables in each model to examine whether the effects of these variables differed between the two study years. The results were largely consistent with those of the original models, in which year and its interaction terms were excluded. However, we also detected several significant effects of year and observed notable interaction patterns. Specifically, the density of file teeth was higher in the first year at noisy sites, but this pattern weakened in the second year. This interesting pattern also motivated us to perform additional analysis on the number of file teeth (not as the density) and file length; they showed reduced file length and increased file tooth number with noise in the first year. Together, these results therefore suggest that increasing the density of file teeth would be related to both the reduced file length and increased number of file teeth, but this pattern would not be temporally consistent across years, suggesting the developmental plasticity of a possible underlying mechanism of the results, which is consistent with previous findings of elevated frequencies in grasshopper courtship signals in roadside habitats. We have incorporated these results and their implications in the discussion section.

Comment
Line 252: To maintain parity and ease of understanding, please plot file density against pronotum length (Fig. 4).
Response: Thank you for your great suggestion. We have changed two points. First, as you suggested, we have revised the y-unit to density.

Comment
Line 272: Exactly, this is a key point which needs to be tested. Also, are the wing

movement velocities identical between the populations?
Response: Yes, this trait is important to test if the elevated frequencies were observed. We did not measure this but discussed its importance as a future research topic.

##### Comment
Line 311: A bit hand-wavy unless tested.
Response: We have made it nuanced.

##

Reviewer #2 (Remarks to the Author):

##### Comment
Review of the manuscript entitled "Male grasshoppers in roadside habitats have increased stridulatory sound-producing organs" by Jou Yoshigai, Keisuke Nakaoka, Tomoki Hirose, Ryugo Ishida, Daiki Waga, Takehiko Shimizu and Masayuki Senzaki.
Anthropogenic noise threatens the integrity of acoustic communication, and the manuscript examines whether grasshoppers adjust stridulatory morphology and signal frequency in noisy roadside habitats. The study found that male grasshoppers (Glyptobothrus maritimus) from noisy roadside habitats had approximately 3.5% more stridulatory file teeth per unit length compared to those from quiet habitats, providing evidence of morphological adaptation to anthropogenic noise in non-vocal animals. However, despite this morphological change, there was no significant increase in courtship signal frequency, suggesting a decoupling between morphological and acoustic traits. Interestingly, this finding contrasts with previous studies on related species, highlighting that morphological adaptations to noise may not always translate into functional acoustic benefits.

Although the dataset is valuable and the core questions are timely, several aspects of the statistical workflow, figure design, and inferential logic can be improved or clarified:
Response: We appreciate the reviewer's fair assessment of our research. Your constructive comments and suggestions were really helpful for improving our manuscript. Below, we have addressed your concerns carefully.

##### Comment

1. The authors report 306 total individuals (152 noisy and 154 quiet), but the actual dataset contains only 299 individuals (149 noisy and 150 quiet). Did I miss something? Could the authors clarify?

Response: Thank you for catching this. We actually sampled 306 individuals, but 7 individuals lost their hind legs. To avoid confusion, we have decided to mention the samples without these excluded samples in the main text (i.e., we referred to "we collected a total of 299 intact males" in the results).

Comment

2. Seven measurements exceed 15,000 Hz (normal range ~6,000-12,000 Hz). Could they be measurement errors or outliers? If removed, does the trend hold?

Response: Thank you for your feedback. As you suggested, we have implemented additional analysis to check whether we detect the same trend after removing > 15000 Hz data. As a result, we hold the consistent conclusion, but we have adopted the dataset without this frequency range in the revised analysis, as these can be considered outliers.

Comment

3. Although the binary design (Quiet vs. Noisy) is a pragmatic solution, it oversimplifies the actual spectrum of acoustic disturbances in nature. I would strongly recommend including at least one intermediate step – a moderate level - and reanalysing the data. Reclassifying into three ecologically distinct categories may provide clearer biological insights and confirm that the main findings (e.g., morphological changes) persist and are enhanced in interpretability when accounting for moderate noise exposure zones. After inspecting the dataset, I am confident that it has potential. For example - Quiet: ≤ 48 dBA; Moderate: 48 < LAeq < 60 dBA; Noisy: ≥ 60 dBA.

Response: Thank you for your understanding of our approach and for sharing promising feedback to try. While we understand your suggestion, your suggested classification resulted in uneven sample sizes in each category (see the following table). How to classify the noise-level classes is not so straightforward. To address this, we have rerun the models with acoustic environments as a continuous variable, represented by site-level LAeq values. Fundamentally, the new analyses with these continuous values hold consistent results with those with binary variables but we have found more interesting results from the effect of the interaction term between year and noise level. We have replaced all the results sections using the new analyses.

	Quiet	Moderate	Noisy
Peak_data	299	41	269
Morph_data	149	29	121

Comment

4. The authors generated focus stacking images for each body part using Photoshop extensively. Using Photoshop for focus stacking and image analysis could introduce several biases and measurement errors that might affect the reliability of the morphological findings. As far as I know, Photoshop is not considered optimal for focus stacking compared to specialised software (e.g., https://community.adobe.com/t5/photoshop-ecosystem-discussions/focus-stacking-in-photoshop-horrible-results/m-p/8960498). Since automated systems might show some measurement variation compared to manual methods, and the study's key finding is a 3.5% difference, focus stacking, hence, artifacts could potentially account for a substantial portion of the observed difference. To assess whether Photoshop introduced significant bias, I recommend:

- Repeat measurements on a subset using specialised focus stacking software (e.g., zero-cost open-source tools: ImageJ EDF, CombineZP, PICOLAY, etc.; or non-free Helicon Focus, Zerene Stacker; however, Zerene must have a free trial period, if I am not mistaken)
- Compare with direct ImageJ analysis of the same specimens
- Use the raw images and validate the results, if possible

Response: The reviewer pointed out a very critical point worth considering. We also thank the reviewer for sharing the specific way to address the concern. To address it, we have compared the effectiveness of stacking images based on images with a subset of samples using Photoshop and the suggested three software (ImageJ EDF, CombineZP (CZP), PICOLAY). These indicated that Photoshop and CZP provided clear stacking images, and the others did not work well, which resulted in relatively composite stacking images (see below). We therefore focused on the former two software in the next step. Using images synthesized with CZP, we measured the file lengths and compared these values with those obtained by Photoshop. The maximum difference between these two software was 0.03 mm, and the correlation between CZP- and Photoshop-based measurements was high (r = 0.99). Accordingly, we consider the original data based on

Photoshop measurements to be reliable and retained them in the revised analyses.

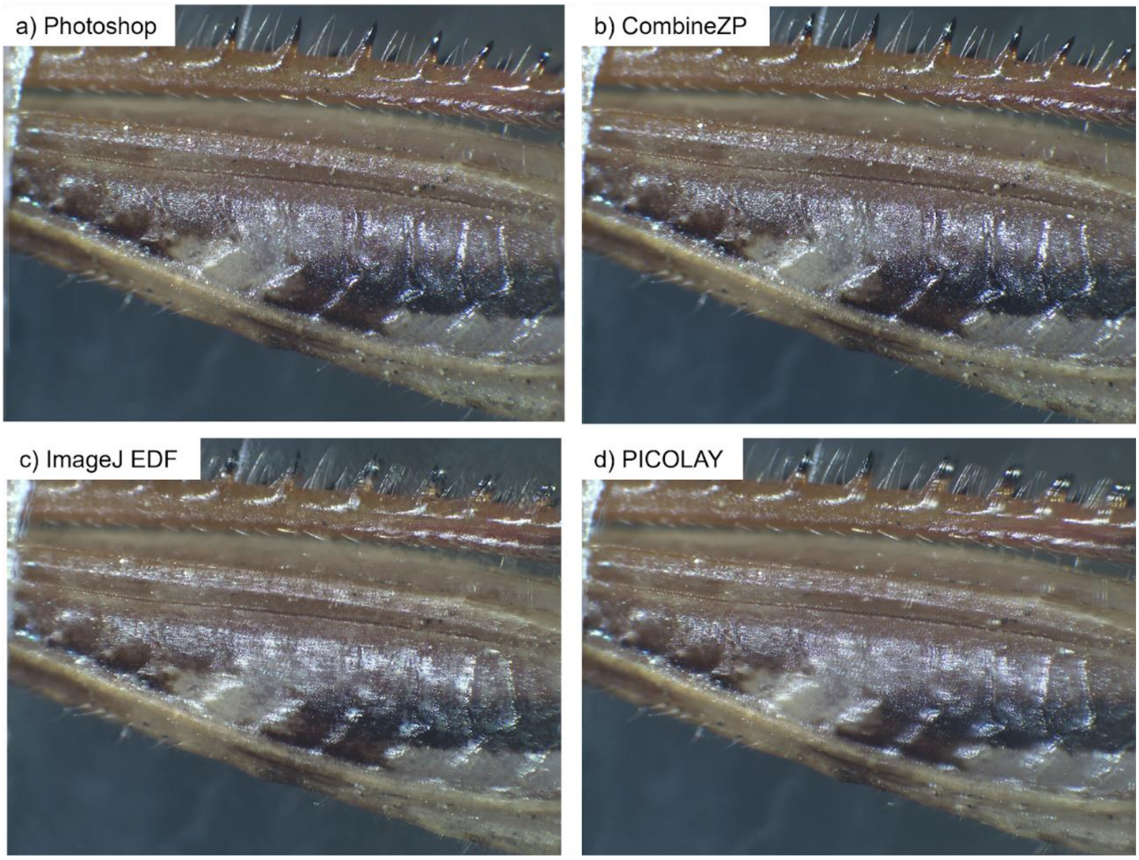

Figure. Comparison of focus stacking images created by the four software. The images show the inner side of the grasshopper's right hind leg.

Comment

5. How meaningful is it to include the year (only two levels) in the models as random factors? To show the temporal consistency between the years, wouldn't it be more meaningful to include the year as fixed effects? It still statistically works this way since not all sites in 2023 were resampled in 2024, and thus there is no perfect overlap between the sites and year factors.

Response: We agree with your suggestion, also commented on by Reviewer 1. We hence included "Year" as a fixed effect. At the same time, we have included interaction terms between year and other variables. The new analysis resulted in a really interesting pattern of the sound-producing organs along a noise gradient; increased file tooth density was evident in the first survey year but not in the second year, suggesting that developmental plasticity would be a key in explaining the results. We have revised the corresponding sections in methods and results, and greatly expanded the discussion about them in the 2nd to 5th paragraphs of the discussion.

Comment

6. Twenty or more individuals in the morphology dataset lack peak frequency measurements. I might have missed that, but could the authors explain?

Response:

Thank you for pointing this out. We carefully re-examined our dataset and found that a total of 23 individuals was lacking in peak frequency data for two reasons: 1) 21 individuals died before signal recording, and thus their courtship signals could not be recorded. 2) For 2 individuals, their signals could be recorded; however, we excluded their sound files due to low sound quality during the acoustic analysis process. Although peak frequency could not be obtained for these 23 individuals, their morphological data were measured because all of them were intact. These individuals were excluded only from the models that included peak frequency as a response variable, thereby avoiding statistical issues. We have added these explanations to the method. We have also added the information about the number of grasshoppers measured in each section of the results.

Comment

7. The authors repeatedly describe the morphological changes as "adaptive responses" without providing sufficient evidence. The authors make strong evolutionary claims based on correlative data. The 3.5% difference in file teeth density could equally result from:

- Developmental plasticity responses to stress
- Population genetic drift in isolated roadside populations
- Indirect selection on correlated traits
- Environmental effects on resource allocation during development

Consider using more cautious language and explicitly discuss alternative hypotheses.

Response: Thank you very much for this very critical comment. First, we have carefully considered whether the use of "adaptive" is suitable or not for each relevant location. Moreover, because new analyses suggested that developmental plasticity would underlie the results, we first discussed the possibility of developmental plasticity rather than genetic differentiation in the 2nd paragraph of the discussion section. We then discussed how this and no corresponding elevation in signal frequency were consistent with the possibility of a maladaptive byproduct of developmental stress in the 3rd paragraph, which was also based on the next comment. Finally, we expanded other possibilities, including indirect selection on correlated traits and environmental effects on resource allocation during development in the 4th paragraph.

Comment
8. The discussion fails to adequately explore that the morphological changes might be maladaptive byproducts of developmental stress. The authors briefly mention this possibility but don't fully develop the argument that increased file teeth without frequency elevation could impose energetic costs without communication benefits. Consider expanding/adding a paragraph in the discussion section.
Response: We appreciate the reviewer's comment and agree with its importance. We have expanded this possibility mainly in the 3rd paragraph of the discussion. See also our response to your 7th comment.

Comment
9. I think the discussion section is narrowly based on two studies of Chorthippus biguttulus. The authors should incorporate the broader literature. For example, focusing on:
- Stress-induced morphological variation in also other insect species
- Developmental constraints on acoustic organ evolution
- Trade-offs between signal production and other fitness components
- Population genetic studies showing neutral evolution of acoustic traits

Response: We are grateful for your suggestion. We have expanded the relevant discussion to include all the possibilities you have indicated in the 4th paragraph of the discussion. According to these revisions, and because we also wanted to value the comparisons between our study and two previous studies of Chorthippus biguttulus, we have largely maintained the comparisons in the original manuscript.

Comment
10. The authors compare Glyptobothrus maritimus with C. biguttulus, focusing primarily on baseline frequencies (10 kHz vs 6-9 kHz), but ignore possible:
- Phylogenetic relationships and evolutionary constraints
- Ecological differences beyond noise exposure
- Methodological differences between studies
- Sample size and statistical power comparisons

Response: Thank you very much for your suggestion. We agree with it. We have added these possibilities.

Comment

11. Unfortunately, the discussion also focuses on oversimplified views of grasshopper acoustic evolution, ignoring recent research on complex genetic architectures underlying acoustic traits and multiple developmental pathways affecting stridulatory morphology, as well as possibly frequency-independent functions of file tooth variation and context-dependent expression of acoustic phenotypes.

Response: The reviewer's view is very valuable to expand the value of the results. We have added a paragraph (i.e., 4th paragraph) that discusses the possibilities you have raised using recent insect literature.

Comment

12. The figures look inconsistent. In Fig. 3, the predicted relationship between pronotum length (x) and peak frequency (y) is plotted, excluding the interaction pronotum x environment. In Fig. 4, however, the interaction is included due to significance. For Fig. 3, consider adding separate-coloured lines (as in Fig. 4) to make the grouping variable visually explicit. Also, plot simple box/violin plots of peak frequency by habitat – I believe this direct visual group comparison is missing and will be expected by most readers.

Response: Thank you very much for your comments. We have improved figure presentations based on your comments, with some modifications, because we have treated acoustic conditions as a continuous noise-level variable and included year and its interaction terms based on your recommendation. We have presented three figures (Figs 3-5), each corresponding to a GLMM with a different response variable. For these, we have presented the relationship between the response variables and noise regardless of the statistical significance, and we have clarified samples from the different years. We have also clarified how the relationship with physiological environmental factors varied between the years in the case of significant interaction terms detected.

Comment

13. I might have missed that, but did the authors investigate possible fluctuating asymmetry (FA)? Statistically, the trait differences the study focuses on (file-tooth density) are therefore unaffected by left-right effects. Consequently, omitting an explicit FA analysis did not bias the main conclusions; however, reporting it would have strengthened the methodological transparency and the discussion section.

Response: Thank you for raising this important matter. As you may know, we have acknowledged this issue and therefore decided to use the file tooth density calculated with the total number of file teeth in both legs (Method). We also checked that the differences

in file number and length between the two legs were concentrated to nearly zero. The reviewer's view would be promising, but our primary interests are the file number and length themselves, and therefore not describing additional discussion about this point at this stage. However, please let us know if you still feel like the discussion is needed.

Comment

14. Consider also reporting P values along with the 90% and 95% confidence intervals. I am personally not a big fan of P values either. However, most readers might still find them intuitive, and I believe it is always better to report more than less.

Response: Thank you for your suggestion. We also reported the P-value in the table of results.

Comment

Minor issues:

15. L24 – Authors mentioned that grasshoppers from noisy sites had a greater number of sound-producing organs… Please note also greater than what, and what is the magnitude (in times or percentages). Please also add values (e.g., percentages) wherever possible. Simply stating “higher” or “lower” is not descriptive (e.g., L114-115).

Response: We have clarified the magnitude here. As for lines 114-115, we have moved this to the results section based on a comment from another reviewer and deleted these unclear words.

Comment

16. L42: Replace “is” with “are”

Response: Done as you suggested.

Comment

17. L229-230: Consider moving this info to the methods section

Response: Thank you very much for this comment. We would like to maintain this because of multiple recommendations from the other reviewers.

Comment

18. L231-232: Consider creating a small table here with reported values

Response: Thank you very much for your suggestion. Because our revised analyses have now been treating nose levels as continuous, and hence it is likely confusing to show morphological measurements between quiet vs. noisy habitats, we have decided to

present this information independent of habitat type (i.e., one metric for one morphological trait) in the most relevant section in the results. We have not created a table for this because the information is minimal.

Comment
19. Consider adding the species name as a keyword, as it is not mentioned in the title.
Response: We have added the scientific name of the species as an additional keyword.

Comment
20. Fig. 2: "G. maritimus" should be in italic
Response: Done as you suggested.

##

Reviewer #3 (Remarks to the Author):

Comment
Manuscript title: Male grasshoppers in roadside habitats have increased stridulatory sound-producing organs.
This research investigates the morphological adaptations in the sound-producing organs of male grasshoppers, specifically in response to anthropogenic noise. The study highlights the potential changes in their courtship signals, emphasising the significance of understanding how environmental factors can influence these vital communication mechanisms, specifically changes in the stridulatory apparatus.

The paper explores a significant topic within the field of ecoacoustics, specifically examining how animal communities respond to anthropogenic activities. Although the impacts of urban noise on bird and bat populations have been extensively studied, research on insects remains limited. Therefore, this study offers insights into one possible adaptation to anthropogenic noise. Nevertheless, the findings do not suggest any changes in sound production structures or the peak frequency of acoustic signals related to anatomical modifications. As such, the authors recommended that future research should focus on behavioural traits.

The findings presented in the manuscript provide a novel perspective; however, the manuscript is not conclusive and fails to cite pioneering work on grasshopper stridulation

to show additional knowledge of the mechanism (see recommended references). The study could also be enhanced by incorporating additional factors. For example investigating behavioural aspects that influence the localisation of the singing individual (such as ground level, grass, cavities, etc.) to improve signal transmission, time of peak activity, or examining social interactions that affect the distribution of males and females would be beneficial. Additionally, a thorough analysis of other acoustic properties besides peak frequency—such as sound pressure levels, calling rates, and song patterns—would further enrich the understanding of the effects of noise on grasshopper communities.
Response: Thank you for your positive evaluation of our work and for giving insightful suggestions. We have learned a lot from your review and have tried to improve our manuscript accordingly. Especially, we have studied the suggested papers and incorporated knowledge from these papers into our discussion. Please see our specific revisions below.

Comment
Specific comments:
1) Road noise is an environmental factor that could influence acoustic communication among grasshoppers. However, the primary focus of the research was on morphological modifications. In our opinion, if road noise does affect the acoustic communication in the examined grasshopper species, one might anticipate initial behavioural adaptations before any noticeable morphological changes occur. Behavioural modifications represent a more flexible response compared to morphological changes. Among these adjustments, grasshoppers may choose to sing from specific locations that enhance sound propagation, such as at ground level, or they may alter their population distribution, resulting in individuals being closer to one another. Did the authors gather descriptive information about the studied populations, such as the number of individuals, the location of singing males, and records of social interactions?
Response: As you understood, the primary scope of the study was to investigate the effects of noise on morphology and acoustic traits in grasshoppers exposed to noise, not community-level response, which we have studied in previous research (e.g., Senzaki et al. 2020 Proc B). We think your thought is promising, and grasshoppers may avoid noisy habitats under certain conditions; however, the density of our target species was high in all study sites, including across noisy sites, likely precluding the possibility of micro-habitat selection to avoid noise exposure within the habitat. We have clarified that our roadside habitats with the target species were well-exposed to traffic noise in the first paragraph of the methods. Based on this fact, we also clarified that *G. maritimus* in noisier

habitats may represent a morphological change in its sound-producing organs in the first paragraph of the methods.

Comment
2) Conversely, the assessment of morphological changes did not consider the hearing organs, particularly the tympanic membranes in females, which serve as the primary receivers of acoustic signals. It is also possible that road noise does not significantly impact the hearing process. Therefore, it would be essential to investigate whether there is any mechanical or neurological filtering mechanism in place that renders road noise irrelevant as an environmental pressure driving adaptive changes. If females were collected, can their tympanic membranes be measured? Additionally, are there records of female responses to noise?
Response: We thank the reviewer for highlighting the importance of examining female auditory membranes. To address this concern, we have now discussed the potential effects of noise on female morphology involved in signal reception in the "Limitations and future research directions" section.

Comment
3) It appears that researchers have gathered valuable data from the communities under study. I would suggest that the authors examine the community structure and the dynamics of changes that occurred throughout the study. This exploration could provide further insights into how road noise affects grasshopper populations, enhancing the understanding of their behaviour and interactions in response to environmental disturbances.
Response: As we responded to your first comment, we think that the reviewer's view is very interesting. However, this aspect is out of scope in the current research and a target of our previous research (Senzaki et al. 2020 Proc B). We therefore decided not to modify the main text about this point.

Comment
Lines 53-53: by stridulating sound-producing organs at relatively constant speeds….
How do you know this? if you see classical papers of von Helversen & Helversen (J Comp Physiol A (1997) 180: 373-386), (Tarasova et al., 2021) The wing motion patterns suggest variable speeds during stridulation. We noticed that you cite Helversen & Helversen (1997), but you did not use it to analyse the stridulatory motion patterns, which suggests variable speeds.

Response: Thank you very much for your suggestion. We have deleted the phrase and modified the sentence a bit to clarify that the necessary behavior may limit their ability to flexibly modulate signal frequencies in noisy environments.

Comment
Line 56-57. You can still have such plasticity without noticeable morphological changes, for example, if the speed increases, causing a high rate of tooth impacts on the stridulatory files.
Response: We agree with this possibility and added a phrase about it here. We have also added this possibility in explaining our results in the discussion section.

Comment
Lines 76-84: all of this here should be placed in methods.
Response: Thank you very much for your suggestion. While we value the reviewer's view, we would like to retain this information in this location because this is necessary guidance to quickly understand the study design. We prefer this style, but please let us know if you still feel that this is not the best.

Comment
Lines 91-95. Do both species live in sympatry?
Response: No, they have allopatric ranges. We have clarified it.

Comment
Lines 113-117: Even if you are trying to demonstrate that the two sites differ in background noise levels, this should be placed in the results.
Response: We appreciate the reviewer's feedback, which is useful to make the readability clear. We have presented this information in the first section of the results.

Comment
Line 131: I'm very sorry, but this sampling rate is too low; you might be underestimating frequencies > 20 kHz. If you see the paper by Lampe et al, the sampling frequency used in their recordings was 192 kHz, especially because the calling song of this species has significant energy between 7 and 45 kHz.
Response: Thank you very much for your comment on the need to validate the used sampling rate. The spectrum of traffic noise is dominated by low-frequency components (mainly <10Hz), and hence higher frequency components of the courtship signals in both

our target and related species are unlikely to be affected by traffic noise. Based on this assumption, Lampe et al focuses on the frequency range between 6-9kHz in their analysis despite the use of a 192 kHz sampling rate, indicating that a higher sampling rate would not be needed and 44.1kHz is enough for our study focus. We have explained this in the "sound recording and analysis" section.

Comment
Lines 133-135: more details here, what was the distance between microphone and the singing insect? did you measure sound levels of the recorded songs? why not?
Response: We agree that SPL is another important trait, although its measurement requires special equipment and technique. This study focused on frequency and designed the experiment to assess the effect of noise exposure on frequency shift. We were therefore unable to record the SPL of the signals. However, to address the concern as much as possible, we have added the information about the distance between the recorder and signaling males.

Comment
Line 143: "peak frequencies of C. biguttulus in the 6-9 kHz frequency…" This is only the dominant frequency, but you are missing a huge amplitude component at about 37 kHz.
Response: The reviewer indicated the important point. As we have responded to the above comment, our interest was most related to a peak in a relatively low frequency band (<15kHz). We have cited a spectrum of courtship signals of our focal species and have clarified this point.

Comment
Lines 156-157: "Body size and sound-producing organs, such as the number and density of stridulatory file teeth…" What about radiator's resonances?
Response: We thank the reviewer for highlighting the importance of resonating structures in sound production. We agree that the resonator plays a crucial role in shaping acoustic signals. In this study, however, we focused on the exciter (i.e., the number and density of stridulatory file teeth), as these traits can be readily and reliably quantified. We have now clarified this rationale in the methods. In addition, we have revised the Discussion to note that explicitly testing the relationship between resonator properties and realized acoustic characteristics under noisy conditions would be an important direction for future research.

Comment

Lines 164-166: Yes, but crickets use a different mechanism; they need control of the carrier frequency to maintain a single tone, and use an escapement mechanism which depends on systemically distributed teeth (among other factors), which will not apply to the grasshoppers as they use broadband signals.
Response: Thank you very much for this comment. We have deleted this sentence because we have now realized that it is not necessary.

Comment
Lines 230-233: This here is confusing. Can you present the stats in a more organised way?
Response: Thank you very much for your suggestion. Because our revised analyses have now been treating nose levels as continuous, and hence it is likely confusing to show morphological measurements between quiet vs. noisy habitats, we have decided to present this information independent of habitat type (i.e., one metric for one morphological trait) in the most relevant section in the results. We have not created a table for this because the information is minimal.

Comment
Figures and table: can you please present the equation parameters: slope, intercepts and other relevant statistics?
Response: We have presented estimates of intercept and explanatory and random variables, 95%CI, P-value, and diagnostics about model fitting in the table. In the figures, we have cited the table and clarified which relationship has been shown and why.

Comment
Lines 239-242: The length of the file in relation to body size has been shown in other stridulating orthoptera, crickets and katydids (Chivers et al., 2017; Montealegre-Z et al., 2017), so it is not surprising that the extra number of teeth correlates with body size. And in relation to this, a coherent expectation would be that longer files are associated with lower frequencies.
Response: Thank you very much for your comment. While we value it, we would like to maintain this result here because this is in the results section, and one may be interested in the same results found in our study, in addition to previous ones. As for the relationship between file length and body size, we agree with that. But our new results have shown that individuals exposed to noise would have shorter file lengths in the first survey year. We have added this result.

Comment

Discussion

Lines 256-259: Following from my comments above, relating to body size, file length, etc, this section deserves a better interpretation. For example, you should focus more on exploring sound levels, under the hypothesis that those insects living in regions exposed to human noise tend to sing louder. For this you will need properly calibrated equipment. In crickets for example, large males tend to be louder (Gray, 1997).

In summary, the authors should provide additional interpretation of the data to produce a more coherent manuscript.

Response: We are grateful for your valuable comment. We agree with the importance of considering the sound level aspect. At the same time, as we have responded to the above comment, our primary focus was on the possibility of frequency shift, and it was also difficult to quantify the SPL precisely. To address the reviewer's concern, we have discussed that responses in SPL of the signal rather than frequency should be examined in future work based on the knowledge of Lombard effects and the suggested paper (Gray 1997).

Comment

Recommended references

Chivers, B. D., Jonsson, T., Soulsbury, C. D. & Montealegre-Z, F. 2017. Structural biomechanics determine spectral purity of bush-cricket calls. Biol. Lett. 13.

Gray, D. A. 1997. Female house crickets,Acheta domesticus, prefer the chirps of large males. Animal Behaviour 54: 1553-1562.

Montealegre-Z, F., Ogden, J., Jonsson, T. & Soulsbury, C. D. 2017. Morphological determinants of signal carrier frequency in katydids (Orthoptera): a comparative analysis using biophysical evidence of wing vibration. J. Evol. Biol. 30: 2068-2078.

Tarasova, T. A., Sevastianov, N. S. & Vedenina, V. Y. 2021. Songs and morphology in grasshoppers of the Stenobothrus eurasius group (Orthoptera: Acrdidae: Gomphocerinae) from Russia and adjacent countries: clarifying of taxonomic status. Zootaxa 4965: 244260.

Response: Thank you very much for letting us know useful references. We have carefully reviewed these papers and cited two of them on relevant areas.

###

Reviewer #4 (Remarks to the Author):

Comment
I co-reviewed this manuscript with one of the reviewers who provided the listed reports. This is part of the Communications Biology initiative to facilitate training in peer review and to provide appropriate recognition for Early Career Researchers who co-review manuscripts.

Response: Thank you very much for supporting one of the reviewers.

Manuscript title: Male grasshoppers in roadside habitats have increased stridulatory sound-producing organs.

General comment:

This research investigates the morphological adaptations in the sound-producing organs of male grasshoppers, specifically in response to anthropogenic noise. The study highlights the potential changes in their courtship signals, emphasising the significance of understanding how environmental factors can influence these vital communication mechanisms.

The paper explores a significant topic within the field of ecoacoustics, specifically examining how animal communities respond to anthropogenic activities. Although the impacts of urban noise on bird and bat populations have been extensively studied, research on insects remains limited. This study offers insights into one possible adaptation to anthropogenic noise; however, the findings do not suggest any changes in sound production structures or the peak frequency of acoustic signals related to anatomical modifications. As such, the authors recommended that future research should focus on behavioural traits.

The findings presented in the manuscript provide a novel perspective; however, the study could be enhanced by incorporating additional factors. Investigating behavioural aspects that influence the localisation of the singing individual (such as ground level, grass, cavities, etc.) to improve signal transmission, time of peak activity, or examining social interactions that affect the distribution of males and females would be beneficial. Additionally, a thorough analysis of other acoustic properties besides peak frequency—such as sound pressure levels, calling rates, and song patterns—would further enrich the understanding of the effects of noise on grasshopper communities.

Specific comments:

1) Road noise is an environmental factor that may influence acoustic communication among grasshoppers. However, the primary focus of the research was on morphological modifications. In my opinion, if road noise does affect the acoustic communication in the examined grasshopper species, one might anticipate initial behavioural adaptations before any noticeable morphological changes occur. Behavioural modifications represent a more flexible response compared to morphological changes. Among these adjustments, grasshoppers may choose to sing from specific locations that enhance sound propagation, such as at ground level, or they may alter their population distribution, resulting in individuals being closer to one another. Did the authors gather descriptive information about the studied populations, such as the number of individuals, the location of singing males, and records of social interactions?

2) Conversely, the assessment of morphological changes did not consider the hearing organs, particularly the tympanic membranes in females, which serve as the primary receivers of acoustic signals. It is also possible that road noise does not significantly impact the hearing process. Therefore, it would be essential to investigate whether there is any mechanical or neurological filtering mechanism in place that renders road noise irrelevant as an environmental pressure driving adaptive changes. If females were collected, can their tympanic membranes be measured? Additionally, are there records of female responses to noise?

3) It appears that researchers have gathered valuable data from the communities under study. I would suggest that the authors examine the community structure and the dynamics of changes that occurred throughout the study. This exploration could provide further insights into how road noise affects grasshopper populations, enhancing the understanding of their behaviour and interactions in response to environmental disturbances.

Review of the manuscript entitled "Male grasshoppers in roadside habitats have increased stridulatory sound-producing organs" by Jou Yoshigai, Keisuke Nakaoka, Tomoki Hirose, Ryugo Ishida, Daiki Waga, Takehiko Shimizu and Masayuki Senzaki.

Anthropogenic noise threatens the integrity of acoustic communication, and the manuscript examines whether grasshoppers adjust stridulatory morphology and signal frequency in noisy roadside habitats. The study found that male grasshoppers (*Glyptobothrus maritimus*) from noisy roadside habitats had approximately 3.5% more stridulatory file teeth per unit length compared to those from quiet habitats, providing evidence of morphological adaptation to anthropogenic noise in non-vocal animals. However, despite this morphological change, there was no significant increase in courtship signal frequency, suggesting a decoupling between morphological and acoustic traits. Interestingly, this finding contrasts with previous studies on related species, highlighting that morphological adaptations to noise may not always translate into functional acoustic benefits.

Although the dataset is valuable and the core questions are timely, several aspects of the statistical workflow, figure design, and inferential logic can be improved or clarified:

1. The authors report 306 total individuals (152 noisy and 154 quiet), but the actual dataset contains only 299 individuals (149 noisy and 150 quiet). Did I miss something? Could the authors clarify?

2. Seven measurements exceed 15,000 Hz (normal range ~6,000-12,000 Hz). Could they be measurement errors or outliers? If removed, does the trend hold?

3. Although the binary design (Quiet vs. Noisy) is a pragmatic solution, it oversimplifies the actual spectrum of acoustic disturbances in nature. I would strongly recommend including at least one intermediate step – a moderate level - and reanalysing the data. Reclassifying into three ecologically distinct categories may provide clearer biological insights and confirm that the main findings (e.g., morphological changes) persist and are enhanced in interpretability when accounting for moderate noise exposure zones. After inspecting the dataset, I am confident that it has potential. For example - Quiet: ≤ 48 dBA; Moderate: 48 < LAeq < 60 dBA; Noisy: ≥ 60 dBA.

4. The authors generated focus stacking images for each body part using Photoshop extensively. Using Photoshop for focus stacking and image analysis could introduce several biases and measurement errors that might affect the reliability of the morphological findings. As far as I know, Photoshop is not considered optimal for focus stacking compared to specialised software (e.g., https://community.adobe.com/t5/photoshop-ecosystem-discussions/focus-stacking-in-photoshop-horrible-results/m-p/8960498). Since automated systems might show some measurement variation compared to manual methods, and the study's key finding is a 3.5% difference, focus stacking, hence, artifacts could potentially account for a substantial portion of the observed difference. To assess whether Photoshop introduced significant bias, I recommend:
   - Repeat measurements on a subset using specialised focus stacking software (e.g., zero-cost open-source tools: ImageJ EDF, CombineZP, PICOLAY, etc.;

or non-free Helicon Focus, Zerene Stacker; however, Zerene must have a free trial period, if I am not mistaken)

- Compare with direct ImageJ analysis of the same specimens
- Use the raw images and validate the results, if possible

5. How meaningful is it to include the year (only two levels) in the models as random factors? To show the temporal consistency between the years, wouldn't it be more meaningful to include the year as fixed effects? It still statistically works this way since not all sites in 2023 were resampled in 2024, and thus there is no perfect overlap between the sites and year factors.

6. Twenty or more individuals in the morphology dataset lack peak frequency measurements. I might have missed that, but could the authors explain?

7. The authors repeatedly describe the morphological changes as "adaptive responses" without providing sufficient evidence. The authors make strong evolutionary claims based on correlative data. The 3.5% difference in file teeth density could equally result from:
   - Developmental plasticity responses to stress
   - Population genetic drift in isolated roadside populations
   - Indirect selection on correlated traits
   - Environmental effects on resource allocation during development

   Consider using more cautious language and explicitly discuss alternative hypotheses.

8. The discussion fails to adequately explore that the morphological changes might be maladaptive byproducts of developmental stress. The authors briefly mention this possibility but don't fully develop the argument that increased file teeth without frequency elevation could impose energetic costs without communication benefits. Consider expanding/adding a paragraph in the discussion section.

9. I think the discussion section is narrowly based on two studies of *Chorthippus biguttulus*. The authors should incorporate the broader literature. For example, focusing on:
   - Stress-induced morphological variation in also other insect species
   - Developmental constraints on acoustic organ evolution
   - Trade-offs between signal production and other fitness components
   - Population genetic studies showing neutral evolution of acoustic traits

10. The authors compare *Glyptobothrus maritimus* with *C. biguttulus,* focusing primarily on baseline frequencies (10 kHz vs 6-9 kHz), but ignore possible:
   - Phylogenetic relationships and evolutionary constraints
   - Ecological differences beyond noise exposure
   - Methodological differences between studies
   - Sample size and statistical power comparisons

11. Unfortunately, the discussion also focuses on oversimplified views of grasshopper acoustic evolution, ignoring recent research on complex genetic architectures

underlying acoustic traits and multiple developmental pathways affecting stridulatory morphology, as well as possibly frequency-independent functions of file tooth variation and context-dependent expression of acoustic phenotypes.

12. The figures look inconsistent. In Fig. 3, the predicted relationship between pronotum length (x) and peak frequency (y) is plotted, excluding the interaction pronotum x environment. In Fig. 4, however, the interaction is included due to significance. For Fig. 3, consider adding separate-coloured lines (as in Fig. 4) to make the grouping variable visually explicit. Also, plot simple box/violin plots of peak frequency by habitat – I believe this direct visual group comparison is missing and will be expected by most readers.

13. I might have missed that, but did the authors investigate possible fluctuating asymmetry (FA)? Statistically, the trait differences the study focuses on (file-tooth density) are therefore unaffected by left-right effects. Consequently, omitting an explicit FA analysis did not bias the main conclusions; however, reporting it would have strengthened the methodological transparency and the discussion section.

14. Consider also reporting *P* values along with the 90% and 95% confidence intervals. I am personally not a big fan of *P* values either. However, most readers might still find them intuitive, and I believe it is always better to report more than less.

Minor issues:

15. L24 – Authors mentioned that grasshoppers from noisy sites had a greater number of sound-producing organs… Please note also greater than what, and what is the magnitude (in times or percentages). Please also add values (e.g., percentages) wherever possible. Simply stating “higher” or “lower” is not descriptive (e.g., L114-115).

16. L42: Replace “is” with “are”

17. L229-230: Consider moving this info to the methods section

18. L231-232: Consider creating a small table here with reported values

19. Consider adding the species name as a keyword, as it is not mentioned in the title.

20. Fig. 2: “G. maritimus” should be in italic
